# IID432, a parasite-selective topoisomerase II inhibitor, achieves rapid single-dose parasite clearance in a murine chronic Chagas model

**DOI:** 10.1073/pnas.2620085123

**Published:** 2026-09-09

**Authors:** Manuel Saldivia, Rajiv S. Jumani, Bryanna Thomas, Grace M. Baxley, Jayant Sancheti, Domenico Bullara, Jean-Rene Galarneau, Harry Cheung, Yen-Liang Chen, Reginara Souza DeAsis, Debjani Patra, Olivier René, Jonas Noeske, Andreas D. Schenk, Colin Deniston, Samarth Thakore, Catherine Luu, Charles Wartchow, Dennis C. Koester, Amanda Fortes Francisco, Johanne Blais, Jan Jiricek, Scott A. Hollingsworth, Jonathan E. Gable, John M. Kelly, Natasha Hochberg, Charlie G. Knutson, Suresh B. Lakshminarayana, Christopher Sarko, Ujjini H. Manjunatha, Colin S. Osborne, Thierry T. Diagana, Srinivasa P. S. Rao

**Affiliations:** ^a^Global Health, Biomedical Research, Novartis, Emeryville, CA 94608; ^b^Pharmacokinetic Sciences, Novartis Healthcare Private Limited, Hyderabad 500081, India; ^c^Modeling and Simulation, Biomedical Research, Novartis, Cambridge, MA 02139; ^d^Preclinical Safety, Biomedical Research, Novartis, Cambridge, MA 02139; ^e^Global Discovery Chemistry, Biomedical Research, Novartis Pharma AG, Emeryville, CA 94608; ^f^Molecular Discovery Science, Biomedical Research, Novartis, Emeryville, CA 94608; ^g^Protein Sciences, Biomedical Research, Novartis, Basel CH 4056, Switzerland; ^h^Structural Biologics, Biomedical Research, Novartis, La Jolla, CA 92121; ^i^ London School of Hygiene and Tropical Medicine, London WC1E 7HT, United Kingdom; ^j^Computational Chemistry, Biomedical Research, Novartis, Emeryville, CA 94608; ^k^Translational Medicine, Discovery and Profiling, Biomedical Research, Novartis, Cambridge, MA 02139; ^l^Pharmacokinetic Sciences, Biomedical Research, Novartis, Cambridge, MA 02139; ^m^Pharmacokinetic Sciences, Biomedical Research, Novartis, Emeryville, CA 94608

**Keywords:** kinetoplastid, Chagas disease, topoisomerase, drug discovery, pharmacology

## Abstract

Chagas disease is a neglected disease that affects millions of people from the Latin American region. Current nitroheterocyclic therapies suffer from long treatment duration and safety-related treatment discontinuations. A safe, short course therapy would significantly benefit Chagas patients. Here, we report the comprehensive biological, structural, pharmacodynamic, and pharmacokinetic characterization of IID432, an optimized cyanotriazole with superior potency, favorable pharmacokinetics, and improved safety profile compared to the lead compound CT1. IID432 is a fast-acting, parasite-selective topoisomerase II poison that achieves sterile cure after a single oral dose in a murine model of chronic Trypanosoma cruzi infection. Mechanistically, IID432 stabilizes the parasite TcTopoII-DNA cleavage complex by covalently engaging a parasite-specific cysteine (Cys477), thereby conferring selectivity over the human TOP2A. IID432 displays favorable oral pharmacokinetics, with no off-target activity on human topoisomerases. Brief exposure of IID432 rapidly induces parasite-specific DNA damage and produces sterilizing activity in vitro and in vivo without recrudescence. Together, these findings identify IID432 as a first-in-class, parasite-selective covalent topoisomerase poison with a potential to significantly shorten treatment duration for *T. cruzi* infections.

## Significance

Chagas disease remains one of the most neglected parasitic infections of the Americas, with current treatments requiring months of dosing and frequently abandoned due to side effects. Patients have long needed a shorter, safer cure. Here, we introduce IID432, an optimized small molecule that achieves sterile cure of chronic *Trypanosoma cruzi* infection in mice after just one oral dose. IID432 works through a distinct mechanism: it forms a covalent bond with a cysteine unique to the parasite’s topoisomerase II, trapping the enzyme on DNA and selectively killing the parasite without affecting its human counterpart. This first-in-class, parasite-selective covalent drug candidate offers a realistic path toward dramatically shortening Chagas disease therapy.

Chagas disease, caused by the protozoan parasite *Trypanosoma cruzi*, remains an urgent global health challenge, affecting more than ten million people and accounting for roughly 40,000 new infections and 12,000 deaths annually (1, 2). Current antiparasitic therapies, benznidazole and nifurtimox, require a long treatment duration (8 wk) and are often discontinued due to adverse events (3). Thus, there is an urgent need for shorter, safer, and more effective treatments. Over the last decade, several new antiparasitic agents with novel mechanisms of action have been successfully identified for Chagas disease, showing variable efficacy in preclinical mouse models (4–11). However, achieving complete and durable clearance of *T. cruzi* parasites in chronic murine models has been a significant challenge, highlighting critical gaps in the development of effective therapies. We previously reported a cyanotriazole (CT) class of antiparasitic agents that selectively inhibit *Trypanosoma* topoisomerase II (TopoII) and achieve rapid cures in murine models of human African trypanosomiasis and Chagas disease (4).

Building on the promising efficacy profile of CT1, subsequent toxicological studies revealed that this compound induces lymphopenia and neutrophilia in rats. Mechanistic investigations attributed these findings to the potential inhibition of sphingosine-1-phosphate (S1P) lyase by CT1, resulting in the accumulation of the bioactive lipid S1P, a key mediator of immune cell trafficking. This liability posed a significant challenge to the compound’s further development. Informed by these insights, a focused medicinal chemistry campaign led to the discovery of IID432, an optimized aminopyrrolidine scaffold that replaced the aza-isoindoline core of CT1 (12). This crucial modification successfully eliminated the S1P-related liability while maintaining balanced favorable physicochemical and pharmacokinetic (PK) properties. Herein, we describe the biological profile, the structural rationale, and PK-pharmacodynamic (PD) properties of IID432, a next generation, highly selective *T. cruzi* TopoII (Tc TopoII) poison. IID432 demonstrated potent on-target parasiticidal in vitro activity and achieved a single-dose, relapse-free parasite clearance in a murine infection model of chronic Chagas disease, with favorable PK properties in both rodent and nonrodent species. These profiles support further progression into clinical development.

## Results

### IID432 inhibits *T. cruzi* Growth, Acting as Selective TopoII Poison.

**Fig. 1.**
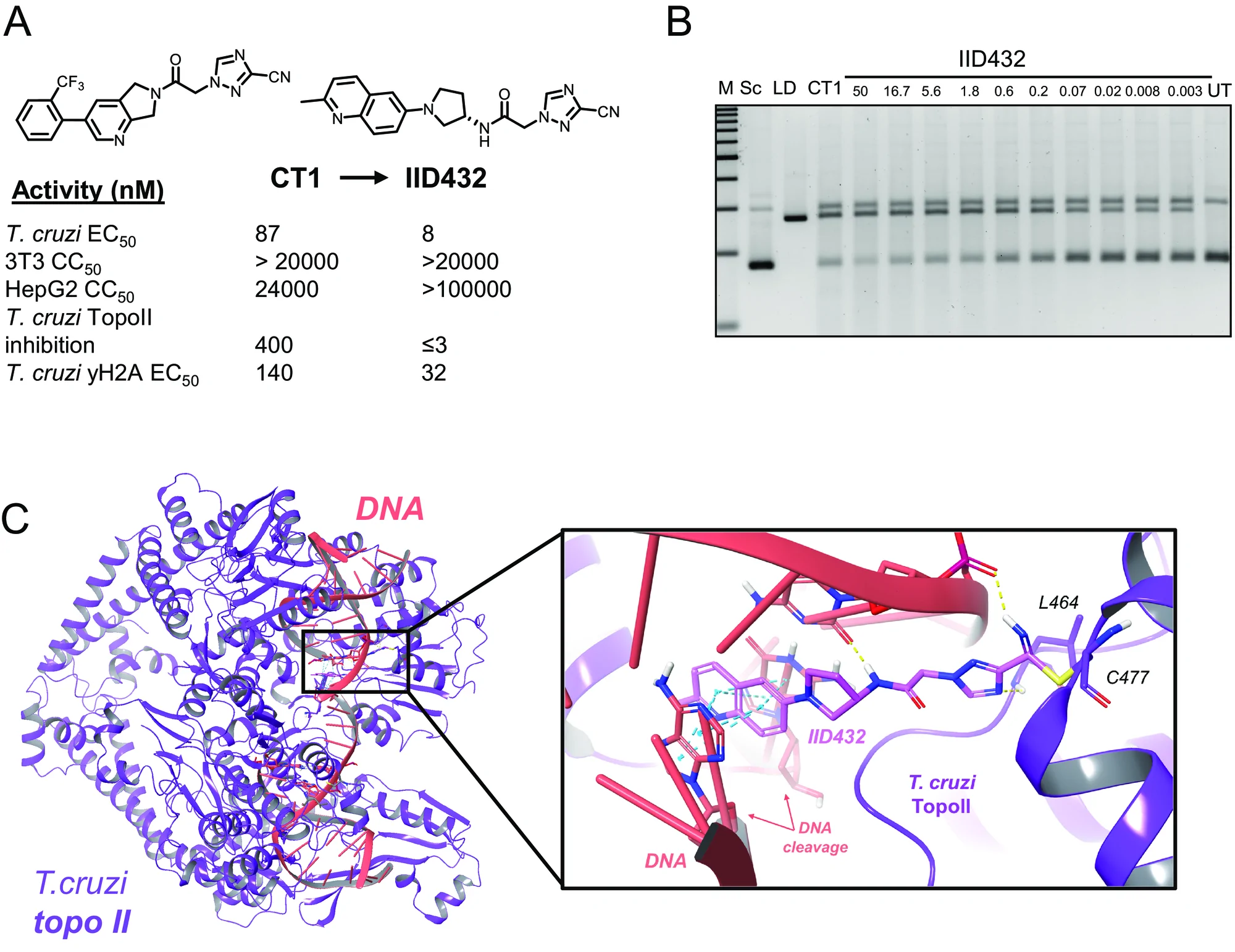
IID432 is a potent *T. cruzi* Topoisomerase II inhibitor. (*A*) Chemical structures and in vitro potency of CT1 and IID432. EC_50_ and CC_50_ values represent the half-maximal growth inhibition concentrations against intracellular *T. cruzi* and mammalian cell lines (3T3 fibroblasts and HepG2 hepatocellular carcinoma), respectively. Mechanism-linked potencies are reported as Topo II inhibition (biochemical DNA linearization) and γH2A EC_50_ (cellular induction of phosphorylated histone H2A in *T. cruzi* following 24-h incubation). Growth inhibition and biochemical measurements were derived from at least two independent biological replicates, each performed in technical duplicates. All values are expressed as geometric means. (*B*) Biochemical characterization of IID432 using a DNA cleavage assay: full-length Tc TopoII was incubated with supercoiled DNA (ScDNA) in the presence of increasing concentrations of IID432, and double-strand DNA break formation in the absence of ATP was assessed by 1.2% agarose gel electrophoresis demonstrating that IID432 results in increased linear dsDNA levels across all concentrations tested. Biochemical experiments were performed twice independently, one of the representative images is shown. M: DNA ladder; Sc: supercoiled DNA; LD: linearized DNA; CT1: full-length TcTopoII incubated with ScDNA in presence of 50 µM of CT1 as positive control; Concentration of IID432 ranged from 50 to 0.003 µM; UT: untreated, Tc TopoII incubated with ScDNA with DMSO only (*C*) Cryo-EM structure of IID432 stabilizing the Tc TopoII–dsDNA cleavage complex through covalent bond formation between the cyanotriazole moiety of IID432 and Cys477, with IID432 (pink) bound to Tc TopoII (purple) and DNA (red).

IID432 exhibited substantially improved intracellular growth inhibition potency against intracellular *T. cruzi* (EC_50_ = 8 nM) relative to the prior cyanotriazole lead **CT1** (EC_50_ = 87 nM) (4), with no measurable cytotoxicity in HepG2 or 3T3 fibroblasts at the highest tested concentrations (Fig. 1*A*). Rate-of-kill assays indicated concentration and time-dependent cidal activity (*SI Appendix*, Fig. S1*A*). In standard in vitro washout assays, IID432 achieved relapse-free parasite clearance (sterile cure) at 40 nM (5× EC_50_) with 4-d exposure and at 20 nM (2.5× EC_50_) with 8-d exposure, indicating that longer exposure reduces the concentration multiples required to achieve sterile cure (*SI Appendix*, Fig. S1*B*). IID432 demonstrated drug-dependent linearization of double-stranded DNA at significantly lower concentrations (≤0.003 µM, Fig. 1*B* and *SI Appendix*, Fig. S2*A*) compared to CT1 [0.4 µM (4)] in a gel-based cleavage assay. Selectivity profiling against human topoisomerases revealed no detectable inhibition of TOP1, TOP2A, or TOP2B up to 100 µM (*SI Appendix*, Fig. S2 *B*–*E*). Further structure determination using cryoelectron microscopy (*SI Appendix*, Table S1 and Fig. S3) captured a drug-stabilized dsDNA–Tc TopoII cleavage complex in which IID432 intercalates between the DNA base pairs at the cleavage site, while the cyanotriazole warhead forms a reversible covalent interaction with Cys477, consistent with the mechanism established for CT1 (Fig. 1*C* and *SI Appendix*, Fig. S4). The enhanced cellular potency of IID432 is plausibly explained by additional π-stacking interactions made by its quinoline side chain (two conjugated ring systems versus one for CT1’s phenyl ring), together with an extra direct hydrogen bond from IID432’s amide nitrogen to a nearby cytosine base (*SI Appendix*, Fig. S5). Additionally, the quinoline side chain of IID432 extends farther toward the DNA religation site and the catalytic Tyr775, thereby further stabilizing the dsDNA-TopoII cleavage complex (*SI Appendix*, Fig. S6*A*). These interactions collectively stabilize the cleavage complex, resulting in DNA scission as observed in the experimental structure. Tc TopoII has a binding affinity of 1.6 nM for immobilized 36-base pair dsDNA in the presence of IID432, while binding in the absence of compound is minimal as measured by surface plasmon resonance (*SI Appendix*, Fig. S6*B*). Collectively, these data demonstrate that cyanotriazoles are potent and selective Tc TopoII poisons that bind to a region analogous to etoposide in human TOP2A and fluoroquinolones in bacterial gyrase (*SI Appendix*, Fig. S6*C*). The parasite specificity of IID432 likely derives from reversible covalent engagement with Tc TopoII Cys477, which is an asparagine in mammalian TOP2A (*SI Appendix*, Fig. S7). The reaction between a nitrile and a thiol nucleophile to form a thioimidate is considered as a reversible reaction with the energy barrier for the reverse reaction typically not exceeding 23.5 kcal/mol (13).

### IID432 Kills Parasites by Stabilizing Selective dsDNA–TcTopoII Cleavage Complex.

**Fig. 2.**
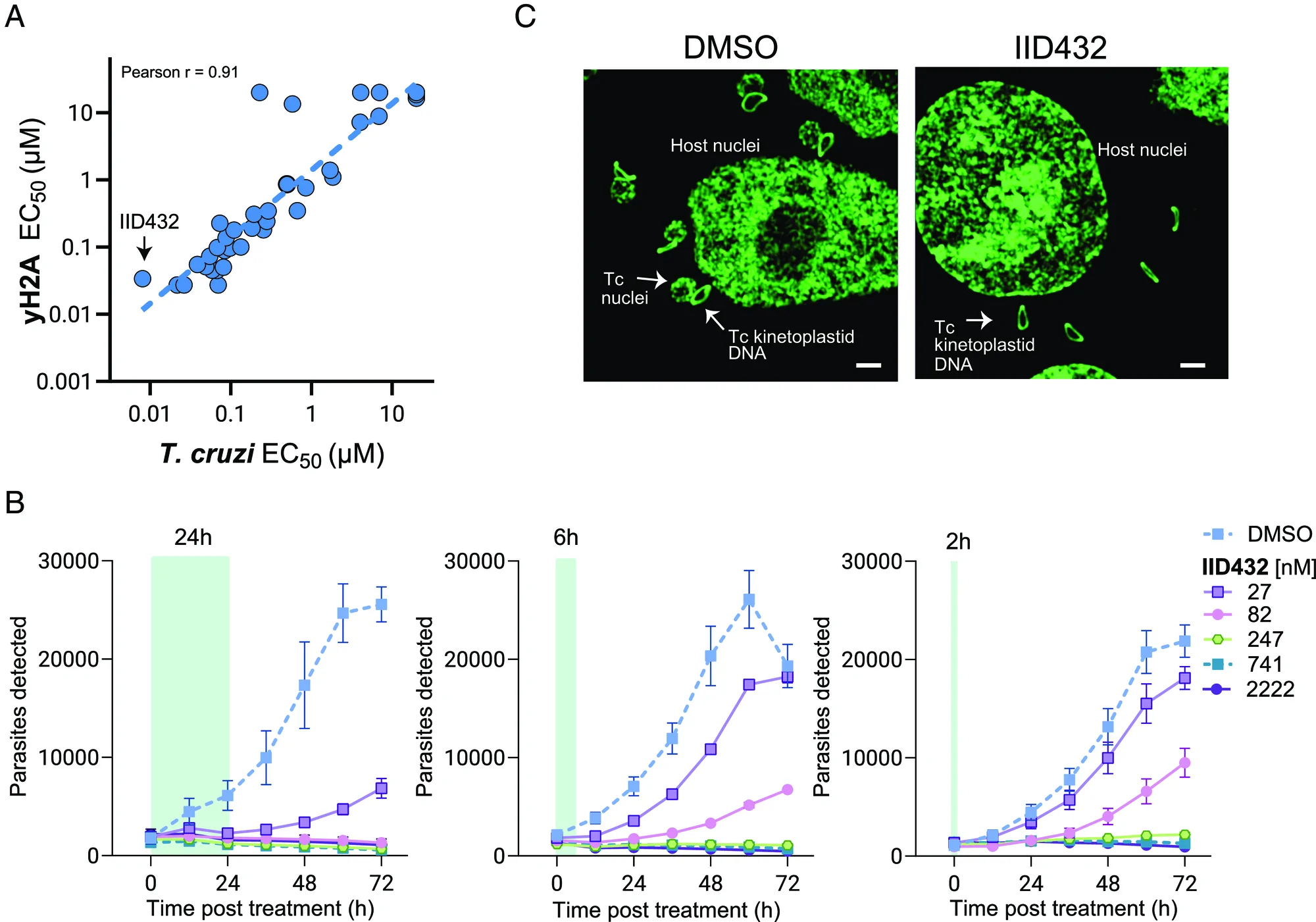
CTs induce *T. cruzi* DNA damage, selectively inhibit nuclear DNA synthesis, and rapidly suppress parasite growth. (*A*) Half minimal concentration required to induce DNA damage as measured by γH2A induction in *T. cruzi* treated with various CTs. Vero cells were infected with *T. cruzi* for 48 h, followed by 24 h of CT compound treatments. Significant correlation (Pearson r = 0.91; *P* < 0.0001) of γH2A EC_50_ with *T. cruzi* parasite growth inhibition EC_50_ was observed. (*B*) Live microscopy imaging of intracellular *T. cruzi* parasites. Vero cells were infected with *T. cruzi* for 24 h, followed by 24, 6, or 2 h of IID432 treatment, after which compound was washed off and intracellular parasite growth was monitored using live microscopy. Data are mean and SD of two or more replicates and are representative of at least three independent experiments. (*C*) Evaluation of active DNA synthesis by using EdU incorporation in *T. cruzi* parasites. Vero cells were infected with *T. cruzi* CL-Brener tdTomato strain for 48 h, followed by 2 h of treatment with IID432 (20 µM) or DMSO. Representative images of intracellular *T. cruzi* with EdU-labeling (green) are shown. IID432 inhibited incorporation of EdU into parasite nuclear DNA but not kinetoplastid DNA or host DNA. Images are representative of at least three independent experiments. (Scale bar, 2 µm.)

Based on the structural data, we hypothesized that IID432 stabilizes the Tc TopoII-dsDNA cleavage complex, triggering DNA damage in *T. cruzi* thereby leading to parasite death. To test this, we quantified DNA damage response to cyanotriazoles using γH2A, a marker of double-strand breaks (14, 15) (*SI Appendix*, Fig. S8*A*) A 24-h treatment with IID432 elicited a robust γH2A signal in *T. cruzi*, and the γH2A EC_50_ values correlated significantly with growth-inhibition EC_50_ values for cyanotriazoles (Fig. 2*A*). Time and concentration profiling showed that IID432 induced γH2A in >25% of parasites as early as 2-h at 0.741 µM (*SI Appendix*, Fig. S8*B*). Building on this rapid DNA-damage response, we evaluated if shorter drug exposures could still achieve parasite growth inhibition after drug washout. Live-cell microscopy with a 24-h exposure followed by drug washout showed that an IID432 concentration of 0.082 µM was sufficient for growth inhibition; 6-h and 2-h exposures required 0.247 µM and 0.741 µM, respectively, to achieve the same outcome (Fig. 2*B*). Notably, these concentrations align with those that produce >25% γH2A positivity (*SI Appendix*, Fig. S8*B*), consistent with a mechanism in which IID432 acts as a TcTopo II poison, stabilizing the TopoII-dsDNA cleavage complex and triggering lethal DNA damage. Complementary studies of DNA replication demonstrated that a 2-h treatment effectively prevents selective incorporation of EdU into parasite nuclei, indicating acute inhibition of nuclear DNA synthesis, but not that of kinetoplastid DNA. No effect on host DNA synthesis was observed (Fig. 2*C*). Cellular studies indicate that shorter treatments require higher concentrations of IID432 to cause lethal levels of irreversible DNA damage in parasites. The same outcome could be achieved with lower IID432 concentrations and longer duration, alluding to a concentration-dependent effect. Together, these results suggest that Tc TopoII inhibition has potential for shortening treatment duration to achieve parasite clearance.

### IID432 Enables Single-Dose Parasite Clearance in Murine Chronic Chagas Disease Model.

In light of the potent in vitro activity exhibited by IID432, its efficacy was evaluated in a highly sensitive bioluminescent mouse model of chronic *T. cruzi* infection (16, 17). Approximately 100 d post infection, mice received oral administration of IID432 at doses ranging from 1 to 60 mg/kg twice daily for 10 d (1, 3, 10, 30, and 60 mg/kg). Except for 1 mg/kg, all doses achieved parasitological clearance, as confirmed by both whole-body and ex vivo organ imaging. Furthermore, a once-daily regimen of 10 mg/kg for 10 d also achieved complete clearance of parasites in all mice after three rounds of immunosuppression, as measured by bioluminescence imaging (BLI) (*SI Appendix*, Table S2 and Fig. S9). In comparison, while the treatment with frontline drug benznidazole (BZN) achieved cure at a dose of 100 mg/kg once daily for 10 d, only 66.7% of mice were cured when 30 mg/kg was given twice daily for 10 d.

**Fig. 3.**
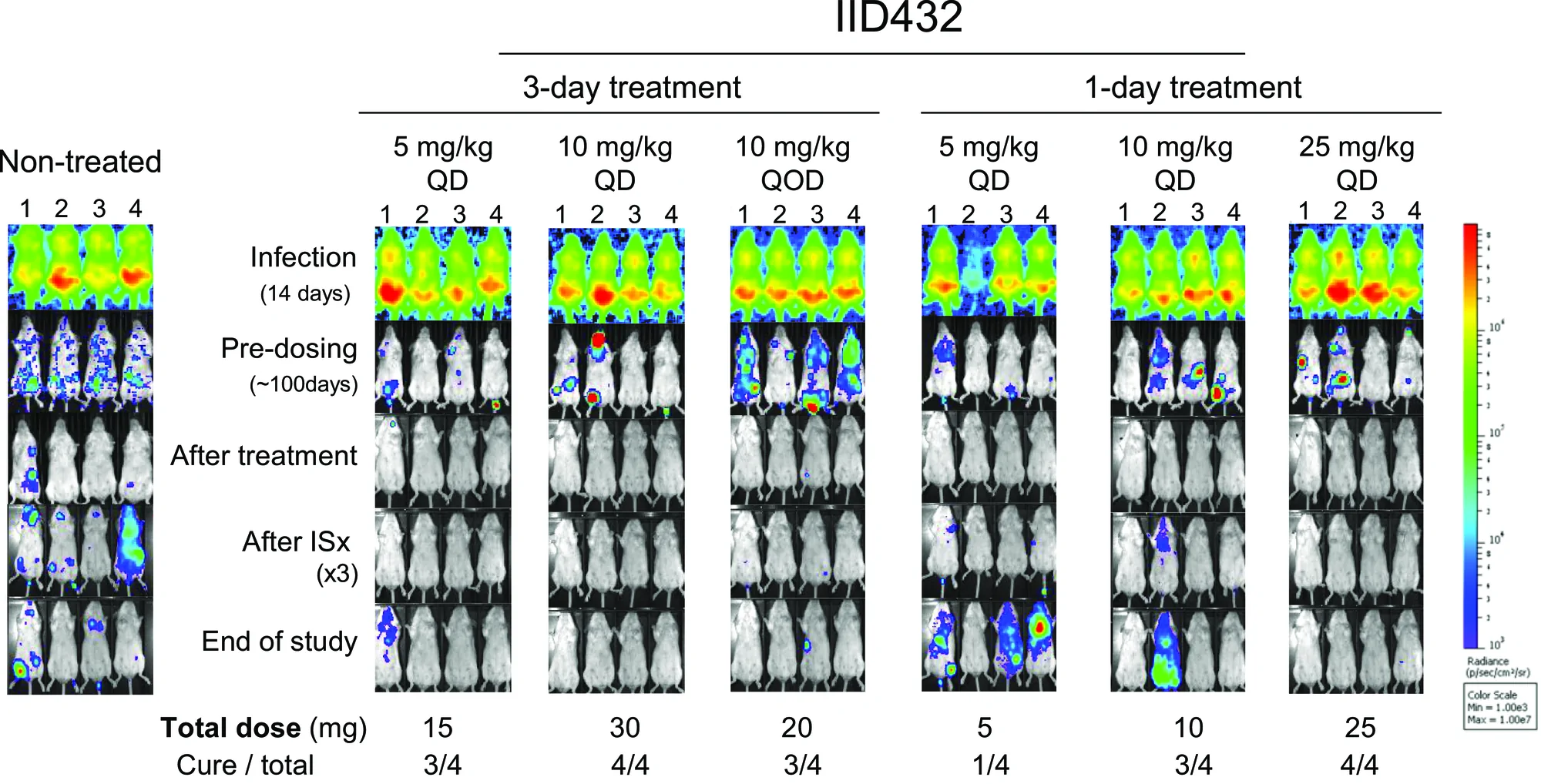
IID432 rapidly clears parasites in a murine model of chronic Chagas disease. BALB/c mice chronically infected with *T. cruzi* were treated with IID432 using different dosing regimens starting at ~day 100 postinfection. Ventral bioluminescence images show longitudinal parasite burden in individual mice (n = 4 per group) at key time points: acute infection (day 14), predosing (~day 100), after treatment, following three rounds of immunosuppression (ISx), and at the study endpoint (day 136). Treatment groups include 3-d regimens (5 mg/kg QD, 10 mg/kg QD, and 10 mg/kg QOD) and 1-d regimens (5, 10, and 25 mg/kg QD). Nontreated infected mice are shown as controls. All treated mice were immunosuppressed with cyclophosphamide (on days 114, 118, and 122) to assess parasite recrudescence. Total administered dose and cure rates (number of mice without detectable bioluminescence/total) are indicated below each group. Individual mice were tracked across time points. QD, once daily; QOD, once every other day.

One of the key objectives of ongoing Chagas disease clinical trials is the development of shorter and safer therapeutic regimens to improve both patient safety and treatment adherence using standard of care nitroheterocyclics (18–21). Building on the rapid, time- and concentration-dependent growth inhibition observed for IID432 in vitro, and its demonstrated ability to achieve complete parasitological clearance with a 10-d dosing regimen, we examined whether comparable efficacy could be attained with shorter in vivo dosing schedules (Fig. 3 and *SI Appendix*, Table S3 and Fig. S10). IID432 treatment resulted in parasite clearance in the mouse model at a cumulative dose of ≥25 mg/kg irrespective of the dosing duration. Specifically, administration of 10 mg/kg IID432 given once daily for 3 d (total dose of 30 mg/kg) resulted in parasitological cure in all four mice. A single dose of 25 mg/kg also achieved complete parasite clearance in all four mice When the total dose was reduced to 20 mg/kg (given as 10 mg/kg once daily, every other day) or 15 mg/kg (given as 5 mg/kg once daily for 3 d) three of four mice achieved parasite clearance (Fig. 3 and *SI Appendix*, Fig. S10). PK analysis of blood samples collected from these studies showed that a 25 mg/kg QD dose, which achieved complete parasitemia clearance, had an exposure [area under the curve (AUC)] of 7,156 ng‧h/mL (*SI Appendix*, Table S4).

To elucidate the relationship between drug exposure and observed efficacy outcomes, a PK/PD analysis was carried out. Additional mouse efficacy studies using varying doses and duration of IID432 treatment were performed to support PK/PD analysis (*SI Appendix*, Table S3 and Fig. S11). A single dose of 25 mg/kg achieved parasite clearance in five out of eight mice. At a single dose of 12.5 mg/kg, 4 out of 8 mice were cured, 5 mg/kg once daily for 5 d cured 3 out of 4 mice and 2.5 mg/kg once daily for 10 d achieved cure in 1 out of 4 mice. Variable efficacies seen in single dose of 25 mg/kg in this study could be attributed to lower drug exposure in mice (*SI Appendix*, Table S4). IID432 was well tolerated and we did not observe any body weight loss during treatment in all mice efficacy experiments with varying doses and dosing regimens.

Population PK modeling using all the infected and naïve mice PK was carried out to derive the PK/PD parameters (*SI Appendix*, Table S5). PK/PD modeling utilizing BLI data also identified cumulative AUC as the principal predictor of cure, in contrast to cumulative time above EC_50_ (*SI Appendix*, Fig. S12). Population PK modeling and simulations established that cumulative AUC above ~8,000 ng‧h/mL in mouse whole blood (corresponds to unbound exposure of ~650 ng‧h/mL in plasma) has potential to achieve clearance of parasites as assessed after immunosuppression. For anticipated human dose projection, with lack of recrudescence, ~650 ng‧h/mL of free plasma cumulative AUC was used as the efficacy benchmark. Collectively, these findings underscore the potential of IID432 to provide highly effective, short-course therapeutic regimens for chronic Chagas disease.

### IID432 Has Favorable ADME Properties in Preclinical Species.

To support the clinical development of IID432, comprehensive in vitro and in vivo ADME (absorption, distribution, metabolism, and excretion) profiling was conducted.

IID432 displayed good aqueous solubility, permeability, low intrinsic clearance in liver microsomes and hepatocytes, good intestinal S9 stability and plasma stability across species (*SI Appendix*, Table S6). IID432 is a highly permeable compound, exhibiting an in vitro permeability of 12.4 × 10^−6^ cm/s in the Madin-Darby canine kidney cell line (MDCK) assay. Plasma protein binding was moderate to high across species ranging from 76.7 to 93.1%. IID432 showed preferential distribution to plasma in mice, rats, and humans. In dogs and nonhuman primates, IID432 was distributed approximately equally between plasma and red blood cells. No meaningful concentration dependence was observed in either the plasma protein binding or blood distribution assessments. IID432 did not exhibit reversible CYP450 inhibition (CYP3A4, 2D6 and 2C9 > 100 μM) or time-dependent inhibition of CYP3A4. In single-donor hepatocytes, IID432 did not induce CYP3A4, 1A2, and 2B6 (*SI Appendix*, Table S6).

The metabolic profile of IID432 was characterized using various human liver-derived systems, including liver microsomes (LM), liver cytosol (LC), and liver S9 (LS9), as well as a recombinant human enzyme. Results indicated good stability of IID432 across these matrixes. Cytochrome P450 (CYP)-mediated biotransformation contributed to the intrinsic clearance of IID432, and there was no indication of UDP-glucuronosyltransferase or sulfotransferase activity. When individual recombinant CYP enzymes were evaluated, contributions were identified from CYP3A4, CYP1A2, CYP3A5, CYP2D6, and CYP2J2 (in descending order) (*SI Appendix*, Fig. S13). CYP3A4 was identified as the principal contributor to IID432 metabolism, accounting for approximately 67.9% of the total CYP mediated activity.

In vitro biotransformation studies on IID432 were conducted using LS9 fractions from rats, dogs, monkeys, and humans, supplemented with NADPH and glutathione to determine metabolic clearance rates and characterize metabolite structures. The clearance of IID432 in LS9 across species was mediated by both GSH-dependent and NADPH-dependent processes. All primary metabolic pathways observed in humans were also seen in preclinical species, with no observed species-specific metabolites (*SI Appendix*, Fig. S14 and Table S7).

In vivo PK analysis of IID432 showed moderate to high oral bioavailability (43 to 88% across species), moderate systemic clearance in rodents and nonhuman primates, and notable high clearance was observed in dogs. The compound also exhibited moderate-to-high volume of distribution (1.1 to 5.7 L/kg) and a short elimination half-life (~1 h) (*SI Appendix*, Table S8).

Human PK parameters were predicted using allometric scaling. Absorption was predicted using a mechanistic model based on physicochemical and in vitro dissolution data, further refined by deconvolution of the monkey absorption profile to account for potential formulation effects. These integrated analyses yielded predicted human clearance values ranging from 1.02 to 1.66 L/h/kg, a steady-state volume of distribution of 3.04 L/kg, and a half-life of 1 to 2 h. The projected oral bioavailability of IID432 ranged from 20 to 53% across a 50 to 1,000 mg dose range. These findings provide a robust foundation for human dose selection and clinical development.

The anticipated human dose for IID432 was projected using a translation modeling approach that combined human PK predictions and mouse PK/PD relations. These human modeling results were consistent with preclinical observations indicating that shorter duration regimens appear clinically feasible and effective. Accordingly, projections of once daily doses of 80 to 140 mg for 14 d or 160 to 300 mg for 7 d are likely to provide clinical benefit.

The preclinical PK and ADME studies of IID432 are favorable and highlight the potential of IID432 for further development as an antiparasitic agent for Chagas disease.

## Discussion

Several clinical trials are being performed to optimize dosing regimens using benznidazole and nifurtimox (18–21). Some shorter dosing regimens have yielded promising sustained parasitemia clearance, as measured by PCR. Despite these advances, challenges such as variable efficacy and tolerability issues remain. Over the last decade, significant progress has been made to discover novel chemical entities for various kinetoplastid pathogens (4–11, 22). These candidates display varying preclinical efficacy in mouse models, with longer treatment duration being a common issue. Several novel preclinical candidates targeting the 20S proteasome and CPSF3 (cleavage and polyadenylation specificity factor 3) are progressing to reach the clinic (Clinicaltrials.gov ID: **NCT07024589**). LXE408, a kinetoplastid selective 20S proteasome inhibitor has advanced into clinical evaluation for both Leishmaniasis (Clinicaltrials.gov ID: **NCT05957978; NCT05593666; NCT06997159**) and Chagas disease (Clinicaltrials.gov ID: **NCT06632600**). Given the high attrition rate from clinical trials to registration, new candidates with novel mechanisms of action are needed. To the best of our knowledge, none of the preclinical candidates have shown single dose cure in a murine infection model of chronic Chagas disease. Extensive medicinal chemistry efforts led to identification of IID432, with improved safety compared to CT1 (12). Inhibition of *T. cruzi* topoisomerase II has shown potential to reduce treatment duration significantly, as demonstrated by IID432. Therefore, IID432 with its promising preclinical profile, exquisite potency both in vitro and in vivo (including single-dose parasite clearance in mice), and favorable nonclinical PK profiles has the potential to shorten antiparasitic treatment for Chagas disease. Further preclinical safety evaluations are in progress. A DNA gyrase inhibitor has contributed to shortening of the treatment period for tuberculosis, as demonstrated by moxifloxacin combination therapy (23). Similarly, IID432, a *T. cruzi* specific TopoII poison, has potential to transform Chagas treatment by considerably reducing treatment duration. Given the treatment-shortening potential of topoisomerase poisons in tuberculosis and Chagas disease, targeting TopoII in other infections could enable rapid pathogen clearance and substantially shorten therapy duration.

## Materials and Methods

Full experimental procedures are provided in *SI Appendix*. This includes i) *T. cruzi* parasite culture, mammalian cell culture, growth inhibition, cytotoxicity, kill-kinetics, washout, live-imaging, DNA-damage, and EdU incorporation assays; ii) biochemical and biophysical characterization of *T. cruzi* topoisomerase II, including cleavage assays, human topoisomerase selectivity assays, and surface plasmon resonance studies; iii) cryoelectron microscopy sample preparation, data collection, image processing, and model refinement; iv) murine acute and chronic *T. cruzi* infection models and BLI studies; v) PK, ADME studies; vi) human PK projection and physiologically based PK modeling; and vii) PK/PD analyses and efficacy benchmarking.

All animal studies were reviewed and approved by the relevant Institutional Animal Care and Use Committees and were conducted in accordance with applicable regulations and institutional guidelines. PK and bioanalytical measurements were performed using validated LC–MS/MS methods.

## Supplementary Material

Appendix 01 (PDF)

## Data Availability

The cryo-EM map is available from Electron Microscopy Databank with accession code EMD-59152 (24). The atomic model has been deposited in the PDB with accession code PDB ID: pdb_000032to (25). All compounds and reagents could be obtained through a material transfer agreement from Novartis by contacting Srinivasa P. S. Rao Email: srinivasa.rao@novartis.com. All other data are included in the manuscript and/or supporting information.
