# Supplementary material for: IID432, a parasite-selective topoisomerase II inhibitor, achieves rapid single-dose parasite clearance in a murine chronic Chagas model
Source: Proc Natl Acad Sci U S A. 2026 Sep 9;123(37):e2620085123. doi: 10.1073/pnas.2620085123 (PMC13578800; doi:10.1073/pnas.2620085123)
Supplement: Supplementary file 1 [file pnas.2620085123.sapp.pdf]

PNAS

Supporting Information for
IID432, a parasite-selective topoisomerase II inhibitor, achieves rapid single-dose parasite clearance in a murine chronic Chagas model

Manuel Saldivia, Rajiv S. Jumani, Bryanna Thomas, Grace M. Baxley, Jayant Sancheti, Domenico Bullara, Jean-Rene Galarneau, Harry Cheung, Yen-Liang Chen, Reginara Souza DeAsis, Debjani Patra, Olivier René, Jonas Noeske, Andreas D. Schenk, Colin Deniston, Samarth Thakore, Catherine Luu, Charles Wartchow, Dennis C. Koester, Amanda Fortes Francisco, Johanne Blais, Jan Jiricek, Scott A. Hollingsworth, Jonathan E. Gable, John M. Kelly, Natasha Hochberg, Charlie G. Knutson, Suresh B. Lakshminarayana, Christopher Sarko, Ujjini H. Manjunatha, Colin S. Osborne, Thierry T. Diagana*, Srinivasa P. S. Rao*

*Thierry T. Diagana; *Srinivasa P. S. Rao
Email: thierry.diagana@novartis.com; srinivasa.rao@novartis.com

This PDF file includes:

Supporting text
Figures S1 to S14
Tables S1 to S8
SI References

Supporting Information Text

Materials and Methods

Cells, parasites, growth inhibition and cytotoxicity assays. Type NIH 3T3 fibroblast cells (ATCC #CRL-1658), HepG2 human hepatoma cells (ATCC #CRL-10741) and Vero cells (kidney epithelial cells extracted from an African green monkey; ATCC #CCL-81) obtained from the American Type Culture Collection (ATCC, Manassas, VA;) were maintained in RPMI 1640 medium (Cytiva HyClone, Logan, UT; cat# SH30027.02) supplemented with 10% heat inactivated fetal bovine serum (FBS; Gibco, Waltham, MA; cat# 10082-147) and 50 IU penicillin/50 µg/mL streptomycin (Gibco; cat# 15070063).

T. cruzi Tulahuen strain constitutively expressing E. coli β-galactosidase was obtained from Fred Buckner, University of Washington, Seattle, WA, USA. T. cruzi CL Brener parasites expressing tandem dimer tomato fluorescent protein (tdTomato) were obtained from Dr. Rick Tarleton (University of Georgia, Franklin College of Arts and Sciences, Athens, GA) (1)

Growth inhibition studies using these parasites were carried out using methods described earlier (2). EC50 refers to the 50% growth inhibition potential of compounds as determined by the sigmoidal dose response values analyzed by HELIOS software.

Cytotoxicity against NIH 3T3 and HepG2 cells was determined as previously described (2). Briefly, Cells were seeded into 384-well plates at 400 cells per well, incubated at 37 °C for 24 h and exposed to threefold serially diluted compounds for 96 h. Cell viability was monitored using a Cell Counting Kit-8. CC50 refers to the concentration of compound required for 50% inhibition of cell proliferation as determined by the sigmoidal dose response values analyzed by HELIOS software.

***T. cruzi* kill kinetics and wash out assays.** These experiments were performed using T. cruzi Tulahuen strain constitutively expressing E. coli β-galactosidase as described earlier with minor modifications (3). Briefly, NIH 3T3 cells ($1x10^6$) were infected with *T. cruzi* Tulahuen trypomastigotes strain at an MOI of 5 ($5x10^6$) in 75 cm^2 vented flasks and incubated at 37°C in 5% CO_2 for 48 h. Following the infection, Gibco™ Trypsin-EDTA (0.05%, Thermo Fisher; cat# 25300120) was used as the de-adhering agent. RPMI-1640 media (HyClone; cat# SH30027.02) supplemented with 10% heat inactivated FBS was added to the cell culture flasks when cells were in suspension. Cells were centrifuged at 1500 rpm for 5 minutes at room temperature, resuspended in warm media, counted with a hemacytometer (2-Chip, Bulldog Bio; cat# DHC-N01) and adjust to a density of $3x10^4$ cells/mL. Approximatively 1.2x103 NIH 3T3 infected cells (50 µL) were seeded in 384-well white bottom plates (Greiner, Mount Joy, PA; cat# 781207) containing 100 nL of test compounds at various concentrations ranging from 25 µM to 1 nM. Each assay constitutes of a set of 6 plates, one plate per time point, incubated at 37°C in 5% CO_2. The time of cell addition was considered time 0. At time points of 0, 24, 48, 72, 96 and 120 h, plates were placed at room temperature for 30 minutes and luminescence was measured using the Beta-Glo® Luciferase Assay System kit (Promega, Madison, WI; cat# E4720) as per the manufacturer's instructions and readings taken using the CLARIOstar microplate reader. The assay quantifies β-galactosidase activity, which is directly proportional to the number of metabolically active *T. cruzi* parasite cells.

For *T. cruzi* wash out assays, NIH 3T3 fibroblasts were infected with *T. cruzi* Tulahuen trypomastigotes at an MOI of 5 in 75 cm^2 flasks and incubated at 37°C with 5% CO_2 for 48 h. Post-infection, cells were detached using 0.05% Trypsin-EDTA, resuspended in RPMI 1640 medium with 10% heat-inactivated FBS, centrifuged, and adjusted to 1×10^4 cells/mL. Infected cells (2.5×10^3 per well) were seeded into 48-well plates and allowed to adhere for 4 h. Test compounds were added at concentrations based on EC_{50} multiples, and cells were incubated for 4 or 8 days with media refreshed every 4 days. After treatment, monolayers were washed and maintained in compound-free media, replaced twice weekly. Cultures were examined by light microscopy (Zeiss, 40×) three times weekly for up to 25 days. For wells without motile parasites by days 10–12, cells were trypsinized and transferred to fresh media for continued observation. Absence of motile parasites by day 25 indicated parasite clearance. On day 25, the presence of intracellular parasites was quantified using the Beta-Glo® Assay. Luminescence was measured on a CLARIOstar plate

reader after 30-minute incubation with reagent, and results were normalized to controls. Relapse was defined as the reappearance of motile trypomastigotes during the 25-day washout. Sterile cure was defined as the absence of both motile and intracellular parasites on day 25.

Biochemical *T. cruzi* TopoII cleavage assay. For *T. cruzi* TopoII cleavage assay, recombinant *T. cruzi* TopoII (80 nM; prepared in-house (3)) was incubated with test compounds in reaction buffer (50 mM Tris-HCl pH 8.0, 150 mM NaCl, 10 mM $MgCl_2$, 500 µM DTT, 0.03 mg/mL BSA) in 384-well plates. After 30 min at room temperature, reactions were initiated by adding supercoiled pHOT-1 DNA (2.5 ng/µL final) and incubated at 37 °C for 90 min. Reactions were stopped with SDS and proteinase K, followed by a 15-min incubation at 37 °C. Products were resolved on 1.2% agarose gels, stained with SYBR Gold, and imaged using a Bio-Rad ChemiDoc system. Band patterns corresponding to nicked, linearized, and supercoiled DNA were used to assess TopoII activity and compound inhibition.

Human topoisomerase assays. Human Topoisomerase assays were performed by Inspiralis. Briefly, the decatenation assays were performed by incubating either 1 U of human TOP2A or TOP2B with 200 ng of kinetoplast DNA (kDNA) in the buffer containing 50 mM Tris HCl (pH 7.5), 125 mM NaCl, 10 mM $MgCl_2$, 5 mM DTT, 0.5 mM EDTA, 0.1 mg/ml bovine serum albumin (BSA) and 1 mM ATP. The total reaction volume and time was 30 μl for 30 minutes at 37 °C. The reaction was stopped by adding 30 μl of chloroform/iso-amyl alcohol (24:1) and 30 μl Stop Dye before being loaded on a 1.0% TAE gel. The electrophoresis was run at 90 V for 90 minutes and stained with ethidium bromide. The human TOP2A cleavage assay was performed by incubating 1 U of human TOP2A in the presence of 0.5 μg of supercoiled pBR322 for 30 minutes at 37 °C in the reaction same buffer mentioned above. The total reaction volume was 30 μl for 30 minutes at 37 °C. The reaction was further incubated with 1 % SDS and 0.5 mg/ml protease K for 30 minutes. The reaction was stopped by adding 30 μl of chloroform/iso-amyl alcohol (24:1) and 30 μl stop dye before being loaded on a 1.0% TAE gel. The electrophoresis was run at 85 V for 2 hours and stained by ethidium bromide.

Human TOP1 relaxation assay was run using 1 U of human TOP1 with 0.5 mg of supercoiled plasmid DNA (pBR322) in the 30 ml containing 20 mM Tris HCl (pH 7.5), 200 mM NaCl, 0.25 mM EDTA in the presence of 5% glycerol and 5% DMSO at 37 °C for 30 minutes in the reaction volume of 30 ml. The reaction was stopped by adding 30 μl of chloroform/iso-amyl alcohol (24:1) and 30 μl Stop Dye before being loaded on a 1.0% TAE gel and run for 2 hours at 90 V before staining the bands with ethidium bromide.All images were captured by GeneGenius (Syngene, Cambridge, UK) and analyzed by GeneTools (Syngene, Cambridge, UK).

Surface plasmon resonance (SPR) assay. DNA preparation for SPR and analysis of Tc TopoII:dsDNA interaction using SPR were performed using protocol described earlier*(3)*. Briefly, for SPR studies the following two custom made DNA oligonucleotides were ordered from IDT: forward: 5' Biotin-CAC GGT GCC GAG GAT AAC AAT GAG CTC ATT GTT ATC CTC GG 3'; reverse: 5' CC GAG GAT AAC AAT GAG CTC ATT GTT ATC CTC GGC ACC 3'. Each oligonucleotide was dissolved in duplex buffer (30 mM HEPES, pH 7.5, 100 mM potassium acetate) to a final concentration of 4 mM. Equal volumes of forward and reverse oligonucleotide were mixed and annealed by heating the mixture to 90ºC for 5 minutes then slowly cooling the mixture to room temperature over a period of 2 hours. This yielded 2 mM annealed dsDNA.

SPR analysis for the determination of K_D, k_a and k_d, were performed with a Biacore 8K instrument in 20 mM Hepes pH 7.3, 100 mM KCl, 10 mM $MgCl_2$, 5% glycerol, 0.05% Tween-20, and 0.5 mM TCEP at 20°C and 100 mL/min flow rate. dsDNA was loaded onto a streptavidin-coated Biacore Sensor Chip SA (GE Healthcare) to 100 RU. TcTopoII (residues 2-1182) binding to dsDNA in the presence or absence of 50 mM of IID432 was measured using a 6 point, 3X dilution series starting at 50 μM TopoII with the single cycle kinetics method according to instrument control software instructions. Data were analyzed using the Biacore Evaluation application to generate affinity constants (K_D), association rate constant (k_a), dissociation rate constant (k_d), and maximum response unit (Rmax).

Structure determination by single-particle cryo-electron microscopy. *Complex formation.* The complex between *T. cruzi* TopoII DNA-binding domain (*T. cruzi* TopoII DBD, amino acids 401-1178), dsDNA (dsDNA (5' GG GAT AAC AAT GAG CTC ATT GTT ATC CC 3') and IID432 was formed by incubating 21.4 μM (or 1.91 mg/mL) *T. cruzi* TopoII DBD in the presence of 75 μM dsDNA and 300 μM IID432 in 20 mM HEPES, pH 7.3, 100 mM KCl, 3 mM $MgCl_2$ for 1 hour on ice. The complex was diluted to 0.5 mg/ml in the above-mentioned buffer keeping the IID432 concentration constant, before applying the sample to the electron microscopy grid.

Sample vitrification. For untilted data collection cryo-EM grids were prepared by glow discharging 200-mesh Quantifoil Cu R 1.2/1.3 grids (Quantifoil, Micro Tools GmbH, Grosslöbichau, Germany) for 90s at 15 mA using a PELCO EasyGlow™ glow discharger (Ted Pella, Redding, CA, USA). 4 µl of complex was applied to each grid before the grids were blotted for 5 s using standard Vitrobot filter paper, Ø55/20mm, Grade 595 (Ted Pella) with a blot force of 25 and plunged into liquid ethane using a Vitrobot MK4 (Thermo Fisher Scientific, Waltham, MA, USA) operated at 4º C and 100 % chamber humidity.

For tilted data collection cryo-EM grids were prepared by glow discharging 300-mesh Quantifoil UltrAu 1.2/1.3 grids (Quantifoil, Micro Tools GmbH, Grosslöbichau, Germany) following as protocol as above.

Data collection. Cryo-EM data were acquired using a Cs-corrected Titan Krios transmission electron microscope (Thermo Fisher Scientific) operated at 300 kV acceleration voltage and a nominal magnification of 75,000x. Tilted data were collected to compensate for the preferred particle orientation visible in the untilted dataset. For collection of both tilted and untilted data a bottom mounted Falcon 4 direct electron detector (Thermo Fisher Scientific) was used in Electron Event Representation (EER) mode with an exposure time of 2.85 s and an exposure rate of 7.5 e/pixel/s at a calibrated pixel size of 0.845 Å/pixel. EPU was configured to record groups of 3x3 foil holes by image shift to speed up data collection

Movies for an untilted dataset were collected using EPU 3.0 (Thermo Fisher Scientific). The defocus range was set from -0.6 µm to -2.0 µm in 0.2 µm increments.

Movies for a 30° tilted dataset were collected using EPU 3.0 (Thermo Fisher Scientific). The defocus range for the central foil hole of the group was set from -1.4 µm to -1.8 µm in 0.2 µm increments.

Data processing. Recorded movies for tilted and untilted datasets were processed in parallel to data acquisition by using CryoFLARE, following the same setup described previously (3). For both datasets the CTF parameters were determined using CryoSPARC PatchCTF.

Particles for both untilted and tilted datasets were picked in CryoSPARC v3.3 using the blob picker with circular and elliptical blobs (100–160 Å). Particles were extracted with a box size of 384 × 384 pixels; for the tilted dataset, particles were subsequently down-sampled to 128 × 128 pixels. Poorly picked particles were removed by 2D classification into 50 classes using default CryoSPARC parameters.

All subsequent model generation, classification and refinement steps were performed in C2 symmetry using CryoSPARC default parameters unless otherwise noted. The resulting particle stack from 2D classification was used to generate a single initial model and the model was refined using non-uniform refinement. The refined particles were re-extracted with a box size of 384x384 pixels.

The extracted particles from tilted and untilted datasets were merged and subjected to a heterogeneous refinement in 3 classes, using the volume from the non-uniform refinement as initial reference. The best class from heterogeneous refinement was subjected to a round of non-uniform refinement. This process was repeated in a second round of 3D classification and non-uniform refinement to further improve the refinement.

Local resolution estimation and orientation diagnostics. The local resolution map and orientation diagnostics were calculated based on the final half-maps using CryoSPARC 4.7 with default parameters.

Model refinement. The Alphafold structure of *T. cruzi* TopoII (Uniprot accession code Q4DE53) was used as a starting model for the refinement process. In Phenix dock_in_map was used to initially place the Alphafold model into the EM map35*(4)*.The model was then refined using phenix.real_space_refine with standard settings including secondary structure and NCS restraints. In the first refinement run rigid body refinement was enabled. Grade from Global Phasing was used to generate a restraint file for the ligand, and the ligand was manually placed into the EM density*(5)*. The restraints for the covalent bonds between the ligand and C477, and between Y775 and DNA were generated using ACEDRG(6)

***T. cruzi* DNA damage assay.** Vero cell culture, *T. cruzi* parasite culture and DNA damage assays were adapted from methodologies described earlier*(7)*. Following the standard protocol described, Vero host cells were seeded in 384-well black, clear and flat-bottom microplates (Greiner AG, Ostereich, Austria; cat# 781097) at a density of 5000 cells per well in 40 µL. After allowing the cells to adhere for 30-60 minutes, tdTomato fluorescent *T. cruzi* parasites were added (10 µL) at a multiplicity of infection (MOI) of 5 and plates were incubated for 48 hours at 37°C in 5% CO2. Test compounds (50 nL) were added to obtain final concentrations ranging from 1 µM to 1 nM using a Mosquito LV liquid handler (SPT Labtech, Melbourn, United Kingdom). Following compound addition, plates were incubated for 24 hours in a humidified incubator at 37°C in 5% CO2. To quantify DNA damage, samples were fixed with 4% paraformaldehyde (Electron Microscopy Sciences, Hatfield, PA; cat# 15710-S) and stained following the immunochemistry protocol described earlier (7). The samples were imaged on an ImageXpress Micro Confocal High Content Imaging System (Molecular Devices, San Jose, CA) using a 20x, water objective (0.95 numerical aperture) with a 3x3 site configuration per well (9 sites per well), 50 µm confocal slit, and standard fluorescence filters for DAPI (4′,6-diamidino-2-phenylindole), fluorescein isothiocyanate (FITC), and tetramethylrhodamine (TRITC). Acquired images were exported as TIF files and then analyzed using CellProfiler version 4.2.4 (Broad Institute, Cambridge, MA). Images were analyzed in CellProfiler (Broad Institute) for the number of Vero host cell nuclei, the number of tdTomato parasites, the γH2A foci, and the percentage of tdTomato parasites positive for γH2A. First, images in all channels were analyzed for quality. Blur measurements were calculated on a spatial scale with a window size of 30 and Otsu thresholding with minimized weighted variance and three-classes. Pixels of medium intensity were assigned to the foreground. Next, illumination for each image channel was corrected by subtracting the minimum pixel intensity across the image from size 40 blocks. Primary object identification was then done on each channel for the respective structures. Vero nuclei were identified in the DAPI channel based on size (10-50-pixel units) and minimum cross-entropy thresholding with threshold smoothing scale of 1.3488 in the Hoechst-stained image. TdTomato parasites were identified in the TRITC channel. Parasites were identified by size (4-20-pixel units) and minimum cross-entropy thresholding with a smoothing scale of 1.3488. Distinguishing clumped objects was done based on intensity and dividing lines drawn by propagation. The identification of γH2A foci was done by first masking the FITC channel with the objects (tdTomato parasites) identified in the TRITC channel to remove background and non-specific antibody staining and only identify γH2A signal within tdTomato parasites. Then, γH2A foci were identified in the masked channel by size (2-16-pixel units) and manual threshold of 0.28 and smoothing scale of 1.3488. The percentage of tdTomato parasites positive for γH2A was calculated using the equation 100*(γH2A foci/ tdTomato parasites). Results from Vero nuclei counts, tdTomato parasite counts, γH2A foci counts, and percentage of γH2A positive parasites were exported to a

spreadsheet by CellProfiler. Images were processed through the CellProfiler pipeline and results were formatted in a plate-view format in Excel. Twenty-eight neutral control (NC; DMSO) and active control (AC; 20 µM CT5) samples were present in each plate. The half-maximal effective concentration (EC50) to elicit γH2A response in tdTomato positive parasites was determined using in-plate duplicate 7-point, 3-fold serial dilutions for each compound. Percentage of γH2A positive parasites was plotted against compound concentration, and the EC50 value was calculated using four parameter non-linear regression. Curve fitting was constrained at the top and bottom by the averages of AC and NC, respectively. The reported EC50 values were calculated as µM concentrations using Helios software.

***T. cruzi* live imaging assay.** For the standard live-imaging assay, Vero host cells were seeded in 384-well black, clear-bottom microplates (Greiner AG, Ostereich, Austria; cat# 781097) at a density of 5000 cells per well in 40 µL of complete medium. After allowing the host cells to adhere for 30-60 minutes, tdTomato fluorescent *T. cruzi* parasites in 10 µL of complete medium were added per well at a multiplicity of infection (MOI) of 5. Following parasite addition, compounds and controls were added using a Mosquito LV liquid handler (SPT Labtech, Melbourn, United Kingdom). Parasite growth was then monitored for 96-108 hours by imaging every 12 hours on a Cytation5 live-imaging microscope (Agilent Technologies, formerly BioTek Instruments, Winooski, VT), paired with BioSpa automated incubator (Agilent Technologies, Winooski, VT). Images were acquired using 10X phase-contrast and RFP filter (575- 605 nm wavelengths) and approximately 5 z-stacks. Focus was set using a reference image from a representative NC well.

Washout assays using live microscopy assay. For the live imaging washout assay, plates were seeded with Vero cells and infected with tdTomato *T. cruzi* parasites according to the above cell seeding and infection protocol and then incubated for 18 to 24 hours. Following incubation, the assay plates were treated with DMSO or compounds and incubated for either 2, 6 or 24 hours as indicated. Plates were then washed three times, each with 100 µl of pre-warmed complete media using a MultiDrop dispensing system with the dispensing speed set to slow. In case of bubbles appearing during the removal of media, plates were centrifuged at 130x *g* for two minutes. Following the wash steps, 25 µl of Vero complete media was added to each well, the plate spun at 130 g for two minutes, and then an additional 25 µl of media added to each well. Imaging was then continued on the Cytation5. For control washout experiments, Vero cells were seeded as before, allowed to adhere for at least 30 minutes and then compounds were added at indicated concentrations. After six hours of incubation time, compounds were washed off following the same wash protocol as above and parasites then added at MOI 5, as described earlier.

EdU incorporation assay. Vero host cells (5000 per well in 40 µL of complete medium) were seeded in 384-well glass bottom plates (Cellvis) pre-coated with 50-100 µg/mL of fibronectin (Corning, catalog# 354008) as per the manufacturer's protocol, and infected with tdTomato *T. cruzi* parasites (MOI 5)*(3)*. Following 48 hours of infection, tool and control compounds were added, along with EdU (final concentration, 10 µM). The cells were incubated with EdU for six hours and were fixed with 4% paraformaldehyde (PFA). For staining, the cells were permeabilized with 0.5% Triton X100, washed with phosphate buffered saline (PBS) and stained for EdU using the Click-iT® assay kit (Thermo Fisher Scientific, catalog# C10340) as per the manufacturer's instructions. Samples were then incubated for 15 minutes in Hoechst 33258 (AnaSpec) in 1% BSA (Sigma) in PBS before leaving in PBS and sealing the plates. Plates were imaged using 40x air objective on Zeiss LSM980 using Airyscan technology.

Murine infections and bioluminescence imaging. Female BALB/c mice (6–8 weeks) were housed under controlled conditions (12 h light/dark cycle, 21°C, 30–50% humidity) with ad libitum access to food and water. All procedures were approved by the Institutional Animal Care and Use Committee, Biomedical Research, Emeryville, CA (protocol #2024-059) and by the LSHTM Animal Welfare and Ethical Review Board (UK home office licence PPL P9AEE04E4/PP7589959).

This highly sensitive, imaging-based bioluminescent mouse model enables real-time tracking of *T. cruzi* infection and facilitates the assessment of treatment effectiveness against chronic stage of

infection *(8, 9)*. Briefly, mice were infected intraperitoneally with 1 × 10^3 blood-form trypomastigotes of a luciferase-expressing *T. cruzi* strain, previously propagated in SCID mice. Treatment began either 14 days (acute model) or 14 weeks (chronic model) post-infection. Animals received daily oral doses of IID432, benznidazole, or vehicle control, formulated in 0.5% methyl cellulose and 0.5% Tween-80 in water.

Parasite burden was monitored via in vivo bioluminescence imaging before, during, and after treatment. Mice with low or undetectable bioluminescence underwent immunosuppression using cyclophosphamide administered intraperitoneally (i.p.). The initial dose was 200 mg/kg, followed by up to two additional doses at 3-day intervals. Mice that relapsed prior to completion of the immunosuppression regimen were excluded from further dosing. For in vivo imaging, mice were injected intraperitoneally with D-luciferin potassium salt (200 mg/kg in sterile D-PBS), anesthetized with 2.5% isoflurane in oxygen, and imaged dorsally and ventrally for up to 10 minutes post-injection using an IVIS Spectrum system (Revvity, Hopkinton, MA, USA). For ex vivo imaging, mice were injected with D-luciferin as described above and euthanized 5 minutes later by exsanguination via the axillary vein under terminal anaesthesia (Dolethal solution, Vetoquinol; 200 mg/mL, administered i.p.). Following removal of residual blood, organs, tissues, and the remaining carcass were placed in round or a square Petri dish, soak up with D-luciferin solution (0.3 mg/mL in D-PBS) and imaged immediately for up to 5 minutes. Total flux (photons/s) was quantified using Living Image software v4.8.2, with background signal from uninfected controls used to define detection thresholds *(8, 9)*.

Concurrently, the pharmacokinetic profile of IID432 was assessed in the treated animals by measuring its concentration in the blood of mice administered with various doses and regimens. Bioanalytical evaluation of pharmacokinetic samples was conducted using liquid chromatography-tandem mass spectrometry (LC-MS/MS). PK parameters were derived using a noncompartmental analysis model implemented in Phoenix software, version 8.3.4 (Certara Pharsight, Princeton, NJ). Unbound PK parameters were calculated based on a mouse plasma protein binding rate of 93.1%.

Solubility, permeability and MDR1 efflux assays. Solubility was measured using high through put equilibrium solubility (HT-Eq sol) assays using a miniaturized shake-flask approach and streamlined HPLC analysis as described earlier *(10)*.
The in vitro permeability of IID432 was assessed using the Madin-Darby Canine Kidney Low Efflux (MDCK-LE) cell line in accordance with the standard protocol *(11)*. In addition, MDR1 (P-gp)–mediated efflux was assessed using MDCK-MDR1 cell lines following established standard protocols *(12)*.

In vitro stability assessments. The metabolic stability in liver microsomes and hepatocytes was determined using the compound depletion approach and was quantified by liquid chromatography–tandem mass spectrometry (LC/MS/MS) *(13, 14)*. The assay measures the rate and extent of metabolism as determined by the disappearance of the parent compound, which allows the determination of *in vitro* half-life ($t_{1/2}$), intrinsic clearance (Cl_{int}), prediction of metabolic clearance (CL) and hepatic extraction ratio in various species. Stability assays in intestinal S9 fraction and plasma were performed as previously described *(15)*. Briefly, the metabolic reaction was initiated by addition of compounds to the matrices. Incubation was conducted in a shaking water bath at 37°C, and time points were sequentially taken. Samples were quenched with ice-cold acetonitrile and analyzed by LC/MS/MS. The half-life (t1/2) was calculated from the rate of compound depletion.

In vitro plasma protein binding and blood to plasma ratio. Plasma protein binding of IID432 was determined using an equilibrium dialysis method with a Rapid Equilibrium Dialysis (RED) device. Dialysis incubations were conducted using the RED device placed on an orbital shaker within an incubator maintained at approximately 37 °C and 5% CO_2. At the end of the incubation period, IID432 concentrations were measured in both the donor (dialyzed) and acceptor (dialysate) compartments using an LC–MS/MS method. The *in vitro* blood distribution of IID432 was evaluated by spiking blood with IID432 and incubating the samples at 37 °C with constant agitation on a roller mixer. After incubation, aliquots of blood samples were analyzed by an LC-MS/MS method to

determine IID432 concentrations. The remaining samples were centrifuged to obtain plasma, which was also analyzed by LC-MS/MS for IID432 concentration.

In vitro CYP 450 inhibition and induction. The inhibitory potential of IID432 against major human cytochrome P450 enzymes was evaluated in vitro using human liver microsomes. IID432 was incubated with probe substrates specific for individual CYP enzymes across a range of concentrations to determine reversible inhibition. To assess metabolism-dependent inhibition, pre-incubation of IID432 with microsomes in the presence of NADPH was performed prior to substrate addition. CYP enzyme activity was quantified by measuring metabolite formation, and IC50 values were derived to characterize the inhibition profile.
The CYP induction potential of IID432 (CYP1A2, CYP2B6, and CYP3A4) was evaluated in cultured human hepatocytes from a single donor. Hepatocytes were incubated with multiple concentrations of IID432 for 48 hours, alongside positive controls (omeprazole for CYP1A2, phenobarbital for CYP2B6, rifampicin for CYP3A4) and with vehicle control (0.1% DMSO). CYP induction was assessed by measuring increase in CYP mRNA expression using quantitative real-time PCR.

In vitro metabolic stability in human liver fractions and early CYP phenotyping. The metabolic stability of IID432 was evaluated using pooled human liver microsomes (HLM), cytosol (HLC) and S9 fractions (HLS9) under conditions with and without NADPH, UDPGA, and PAPS. A parent compound depletion approach was applied, with IID432 quantified over time by LC-MS/MS. Time-dependent loss of the parent compound was used to estimate in vitro half-life (t1/2) and intrinsic clearance (CLint), providing an assessment of overall metabolic turnover and cofactor dependence without enzyme specificity.
CYP reaction phenotyping was conducted using recombinant human CYP enzymes to identify the specific CYP isoforms involved in IID432 oxidative metabolism. Following initial screening, time-dependent depletion assays were performed with the most active CYPs in the presence of NADPH, and parent compound concentrations were measured by LC-MS/MS. CYP-specific intrinsic clearance values were derived using the half-life method and scaled to human liver using a relative expression factor (REF) approach to estimate the fraction metabolized (fm) by individual CYP enzymes.

In vitro liver S9 fraction metabolic stability and clearance pathway assessment using liver S9 fractions. The metabolic stability of IID432 was evaluated using a parent depletion approach in liver S9 fractions from rats, dogs, monkeys, and human donors. Incubations were conducted in presence of NADPH and GSH to capture both oxidative and thiol-mediated clearance pathways. Aliquots collected at predefined intervals were analyzed by LC–MS/MS to determine parent compound depletion and assess the clearance across species.
In vivo pharmacokinetic analysis. In vivo pharmacokinetic studies were conducted using non-randomized C57BL6 male mice (n=3), male Sprague Dawley rats (n=3), male Beagle dogs (n=3), and male cynomolgus monkey (n=3). All procedures involving animals were reviewed and approved by the Institutional Animal Care and Use Committees. IID432 was formulated for oral dose administration as a suspension (MC:Tween 80:Water (0.5:0.1:99.4) for mice, rat, dog and MC:Tween80/50 mM acetate buffer, pH 4.0 (0.5/0.1/99.4) for monkeys) and as a solution formulation for intravenous injection (NMP:4%BSA in PBS (10:90) for mice, ethanol/PG/50 mM acetate buffer, pH 4.0 (10/20/70, v/v/v) for rats NMP:PEG 200 (10:90) for dogs and monkeys). The blood samples for pharmacokinetic studies were collected between 0 and 24h post dose. Blood samples were collected into tubes containing K2 or K3 EDTA and kept on ice until centrifugation. These samples were centrifuged within 30 minutes of collection for 10 minutes at ~6000 *g* or 2 minutes at ~9000 *g* in a refrigerated centrifuge (set to maintain at 4°C). The resultant plasma was harvested into fresh tubes placed on ice until transferred to a freezer, set to maintain -60 to -80°C. The concentrations of IID432 at various time points were determined LC-MS/MS. Pharmacokinetic parameters were determined by noncompartmental analysis using Phoenix WinNonLinTM version 8.3 (Certara, Princeton, NJ).

Prediction of human exposure for IID432. The prediction of human PK for IID432 was performed using allometric scaling (single-species and all species), Wajima approach*(16)* and PBPK

modelling. The PBPK model considered absorption based in physicochemical properties as well as formulation-specific characteristics of IID432.

IID432 PK parameters in plasma (CL, Vss), as determined by non-compartmental analysis, for rat, dog, and monkey were scaled to human by allometric scaling*(17, 18)* using Novartis internal PKS Discovery tool v4.8.2. The bodyweight considered for mouse, rat, dog, monkey, and human were 0.025, 0.25, 10, 4, and 60 kg, respectively. The prediction of a human intravenous concentration-time profile was performed by superposition of preclinical intravenous profiles using the steady state concentration (Css)-mean residence time (MRT) approach*(16)*. IID432's oral absorption and resultant F were predicted using the advanced compartmental absorption and transit model in GastroPlus™ (version 9.8).

Efficacy benchmarking and PK/PD modeling. IID432 efficacy was evaluated in two chronic *T. cruzi* mouse studies using photon flux after immunosuppression as the PD endpoint. Whole-blood PK data were modeled with a two-compartment PopPK approach (Monolix), and simulations generated cumulative AUC and time above EC_{50}. Efficacy benchmarks were defined statistically from variability ranges of harmonized photon flux for uninfected versus untreated controls, with the mid benchmark selected for translational use. Mouse blood AUC benchmarks were converted to human plasma AUC under the free drug hypothesis using species-specific blood-to-plasma ratios and free fractions.

Mouse studies and PD assessment. Data were derived from two efficacy studies in BALB/c mice (~25 g) infected for ~100 days prior to treatment. Animals received oral IID432 at varying doses (2.5 – 25 mg/kg), frequencies (QD, QOD), and durations (1-10 days). Post-treatment, mice were monitored for ~30 days and subjected to 1–3 immunosuppression rounds (cyclophosphamide treatments on days 10, 13, and 16 post last dose). Parasitemia was quantified by in vivo imaging (IVIS), with total photon flux after immunosuppression serving as the primary PD endpoint. Mice were also qualitatively classified as cured or relapsed based on whole-body imaging. For PD, photon flux values after immunosuppression were harmonized across experiments by subtracting the median of the uninfected group within each study and adding the median of these medians; the median, interquartile range (IQR), and full range of these harmonized flux values were plotted as vertical summary statistics. For PK, the median, IQR, and range of PK metrics across virtual mice were calculated in each replicate of the in-silico trials performed in the population simulation. The medians across replicates of these statistics were plotted as horizontal summary statistics. Finally, the median, IQR, and full range of harmonized flux values for uninfected and untreated controls pooled across both studies were added as reference marks, consistent with the approach used for individual-level plots.

PK data and PopPK modeling. Whole-blood PK samples were collected at 0.5–24 h post-dose. Data from both studies were pooled and fitted using Monolix 2023R1 with a two-compartment model, linear clearance, and first-order absorption. Oral absorption rate was fixed to reflect rapid absorption. Model acceptance required realistic parameter estimates, identifiable Fisher matrix, and observed data within 90% prediction intervals. Empirical Bayes estimates (EBEs) were generated for individual mice, and population parameters were derived via stochastic approximation expectation-maximization (SAEM).

Simulations and PK metrics. Individual simulations used EBEs to replicate observed dosing schedules; population simulations used SAEM-derived distributions to generate 10 replicate trials of 100 virtual mice each. PK profiles were simulated for 300 h at 0.1 h intervals. Exposure metrics included cumulative AUC and time above EC_{50} (15.5 ng/mL), the latter derived from the in vitro EC_{50} (8 nM) adjusted for IID432 molecular weight, assay free fraction, and mouse plasma binding.

PK/PD relationships and benchmark definition. Photon flux was plotted against exposure metrics for individual mice and simulated populations. Cumulative AUC was selected as the PK driver based on monotonic PK/PD trends. Efficacy benchmarks were defined statistically: a regimen was considered fully curative if its photon flux variability overlapped entirely with that of uninfected

controls. The cumulative AUC threshold was defined as the minimal value such that the range of cumulative AUC values (when accounting for individual PK variability) for all dose groups above this threshold achieved a full cure, corresponding with left edge of the first fully efficacious group.

Translation to human. Mouse blood AUC benchmarks were converted to human plasma AUC assuming equal free drug exposure across species:

$$\mathrm{AUC}_{\mathrm{human,plamsa,total}} = \frac{\mathrm{AUC}_{\mathrm{human,free}}}{fu,p_{\mathrm{human}}B:P_{\mathrm{mouse}}} = \frac{\mathrm{AUC}_{\mathrm{mouse,blood}}}{B:P_{\mathrm{mouse}}} \cdot \frac{fu,p_{\mathrm{mouse}}}{fu.p_{\mathrm{human}}}.$$

This benchmark informed first-in-human dose projections.

Figures

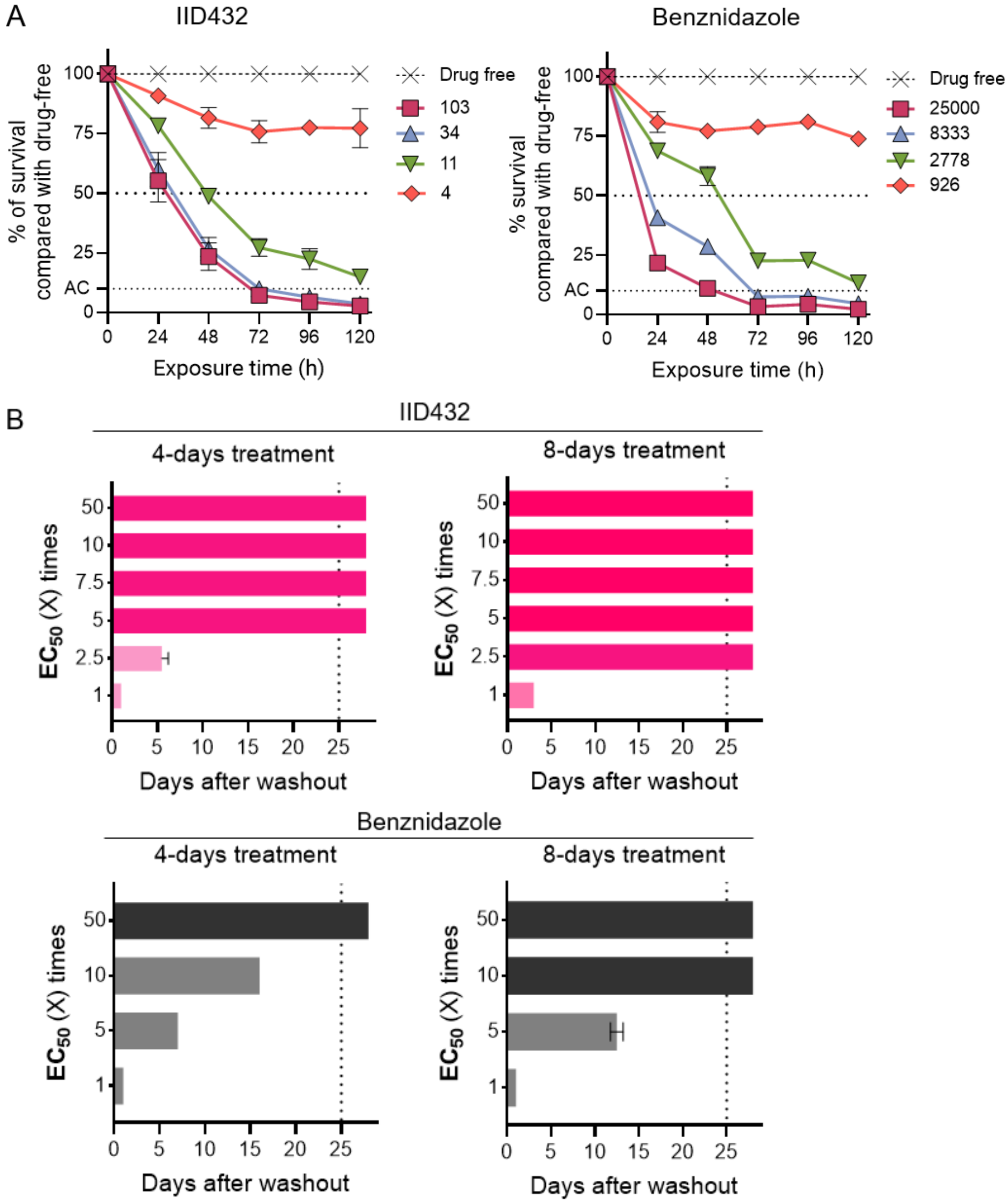

Fig S1. In vitro potency of IID432. (**A**) Time to kill of IID432 and benznidazole using intracellular *T. cruzi* parasites. Data represent the mean ± SEM of three independent biological replicates, where the rate of kill was calculated after comparing each treatment point to a drug-free control over 120 h. An experimental curative benznidazole dose was used as an active control (AC). (**B**) Absolute concentration (ACcure) required of IID432 and benznidazole to achieve sterile cure of intracellular *T. cruzi* parasites after four and eight days of incubation. Data corresponds to three independent in vitro experiments. The absence of motile parasites by live microscopy after 25 days of monitoring was judged to represent lack of relapse.

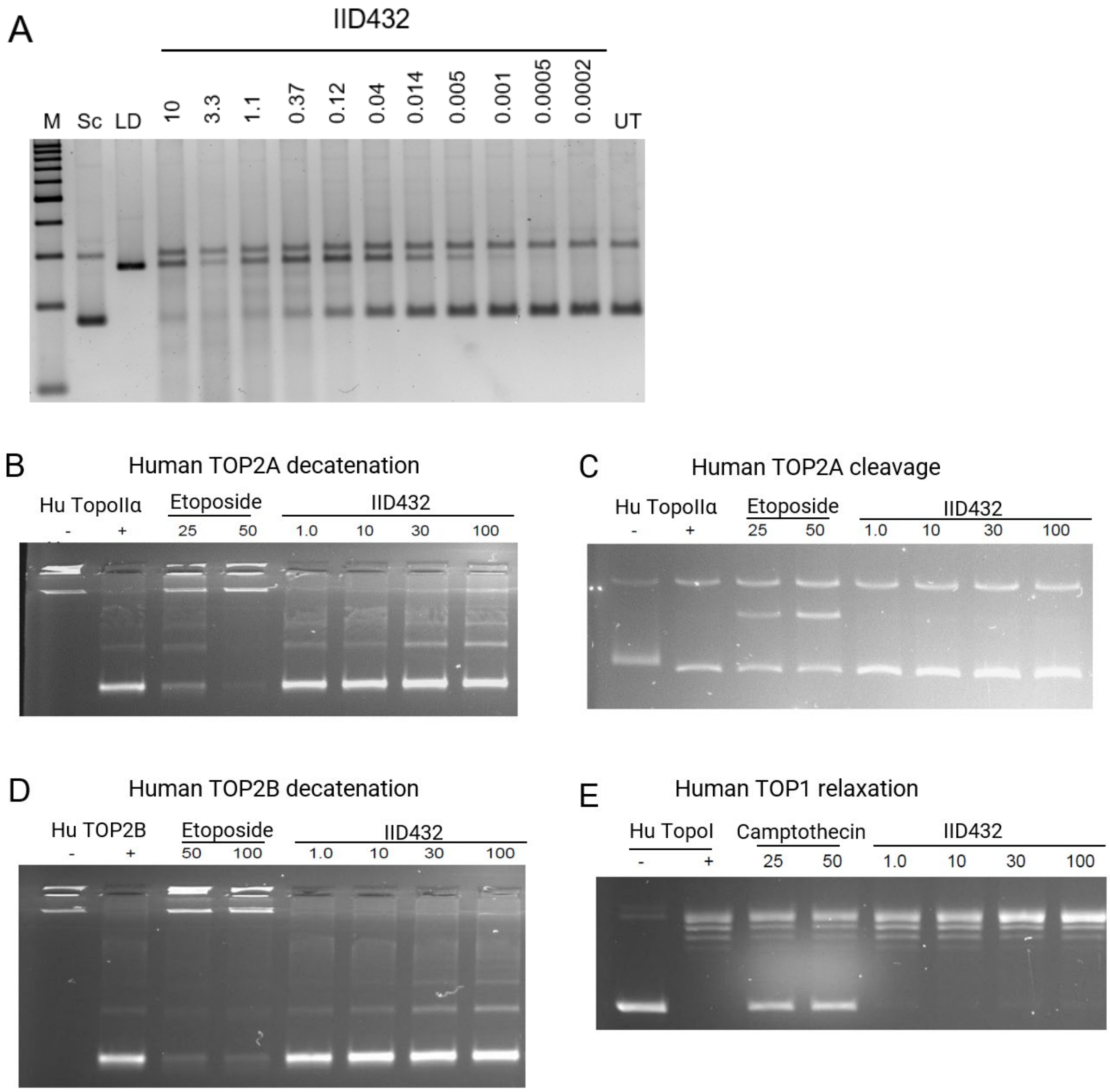

Fig. S2. Biochemical characterization of IID432. (**A**) Inhibition of Tc TopoII DNA cleavage activity by IID432. Full-length Tc TopoII was incubated with supercoiled DNA in the presence of increasing concentrations of IID432, and double-strand DNA break formation in the absence of ATP was assessed by 1.2% agarose gel electrophoresis demonstrating that IID432 results in increased linear dsDNA levels across all concentrations tested. M: DNA ladder; Sc: supercoiled DNA; LD: linearized DNA; Concentration of IID432 ranged from 10 to 0.0002 μM; UT: untreated, Tc topo II incubated with Sc DNA with DMSO only. (**B**) human TOP2A decatenation assay; Kinetoplast DNA (kDNA) was incubated in the presence of Human TOP2A in with Etoposide as positive control (IC_{50} of 23.8 μM). The decatenation resulted in appearance of mini-circular DNA shown on the bottom of the gel. IID432 did not inhibit the decatenation activity Human TOP2A (up to 100 μM). (**C**) human TOP2A cleavage assay; Supercoiled DNA appears at the bottom of the gel in the absence of human TOP2A. The top band represents small amount of nicked DNA. In the presence of human TOP2A, the supercoiled DNA was relaxed, and in the ethidium bromide gel, it ran slightly ahead of the supercoiled DNA (bottom band with human TOP2A). In the presence of DNA poison such as Etoposide (IC_{50} of 33.8 μM), an additional linear DNA band was formed and illustrated as a band in the middle of the gel. IID432 did not appear to inhibit the cleavage activity up to 100 μM. (**D**)

human TOP2B decatenation assay; similar to human TOP2A decatenation assay in figure (A), the positive control Etoposide (IC_{50} of 33.5 μM) inhibited the formation of mini-circular DNA but IID432 did not appear to affect the decatenation activity of human TOP2B up to 100 μM. (**E**) human TOP1 relaxation assay; in the presence human TOPI, supercoiled DNA was relaxed and appears as series of bands representing different relaxation states of the circular DNA. In the presence of camptothecin (IC50 of 37.7 μM), the relaxation activity of TOP1 was inhibited and resulted in the re-appearance of supercoiled DNA. IID432 did not affect the relaxation activity of human TOP1 up to 100 μM. All assays were done in duplicates and the representative gel images was shown with the average IC_{50s} of the positive controls. IID432 did not inhibit human topoisomerases.

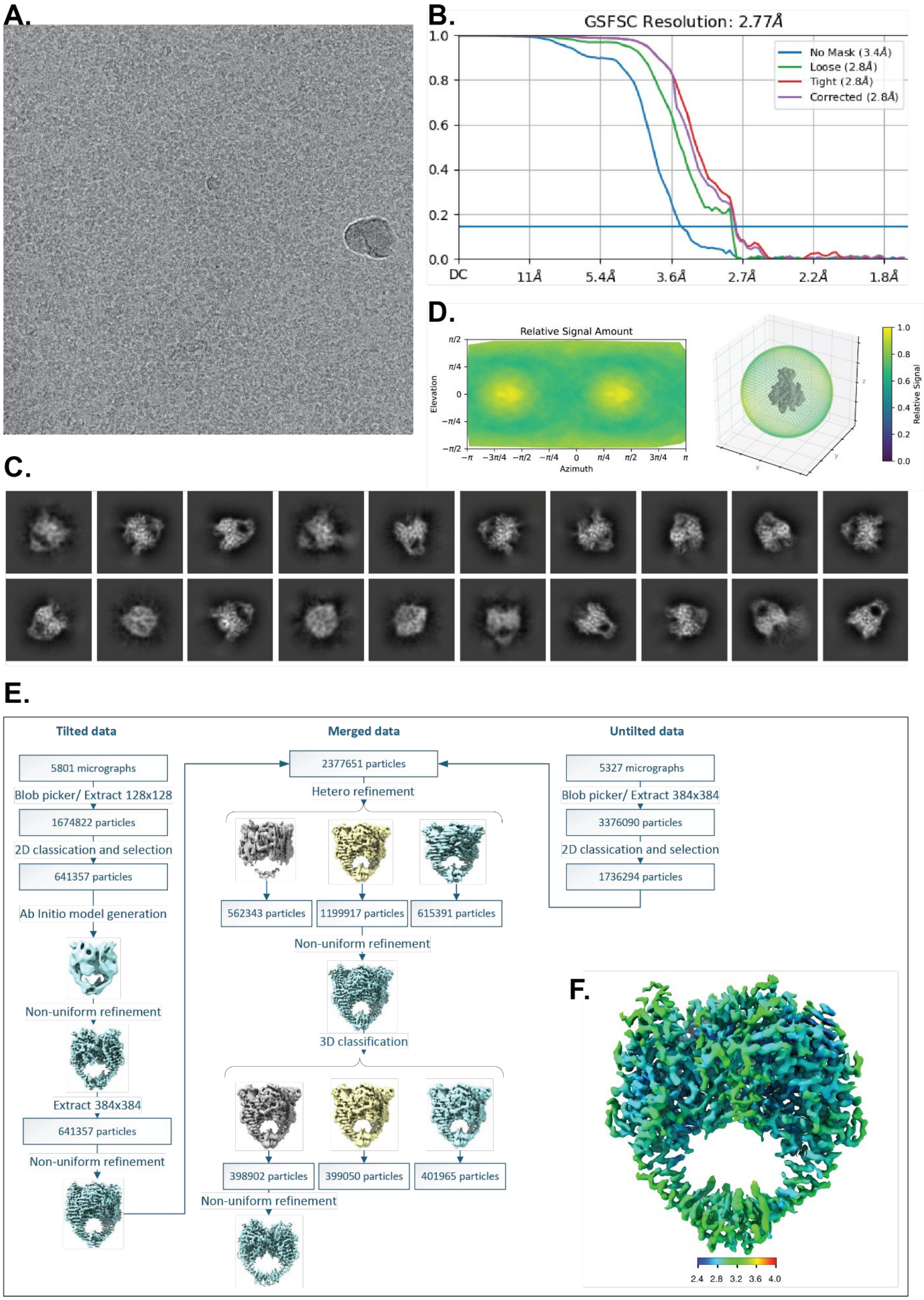
A.
B.
GSFSC Resolution: 2.77Å
No Mask (3.4Å)
Loose (2.8Å)
Tight (2.8Å)
Corrected (2.8Å)
1.0
0.8
0.6
0.4
0.2
0.0
DC
11Å
5.4Å
3.6Å
2.7Å
2.2Å
1.8Å
D.
Relative Signal Amount
Elevation
Azimuth
Relative Signal
C.
E.
Tilted data
Merged data
Untilted data
5801 micrographs
Blob picker/ Extract 128x128
1674822 particles
2D classication and selection
641357 particles
Ab Initio model generation
Non-uniform refinement
Extract 384x384
641357 particles
Non-uniform refinement
2377651 particles
Hetero refinement
562343 particles
1199917 particles
615391 particles
Non-uniform refinement
3D classification
398902 particles
399050 particles
401965 particles
Non-uniform refinement
5327 micrographs
Blob picker/ Extract 384x384
3376090 particles
2D classication and selection
1736294 particles
F.
2.4 2.8 3.2 3.6 4.0

Fig S3. Structure determination of IID432 bound to Tc TopoII and DNA using cryo electron microscopy. (**A**) Representative electron micrograph. (**B**) The final reconstruction shows an average resolution of 2.77 Å, determined by Gold Standard Fourier Shell Correlation with a cutoff of 0.143. (**C**) Representative images of 2D classes. (**D**) Euler distribution plot. Relative signal amount vs. viewing direction as determined by CryoSPARC orientation diagnostics. (**E**) Flowchart of the single particle cryo-EM analysis. (**F**) Local resolution map of TcTopoII.

A

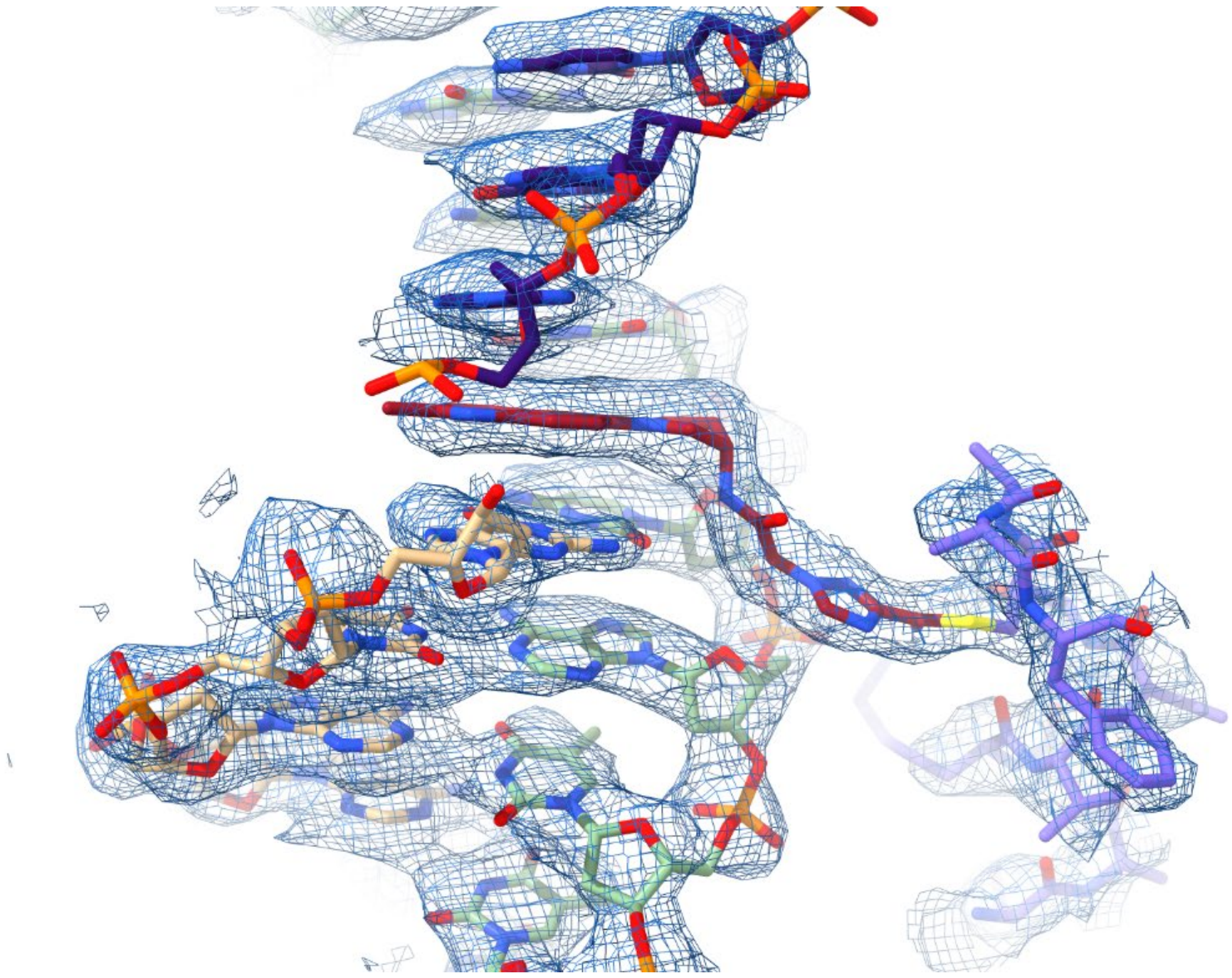

B

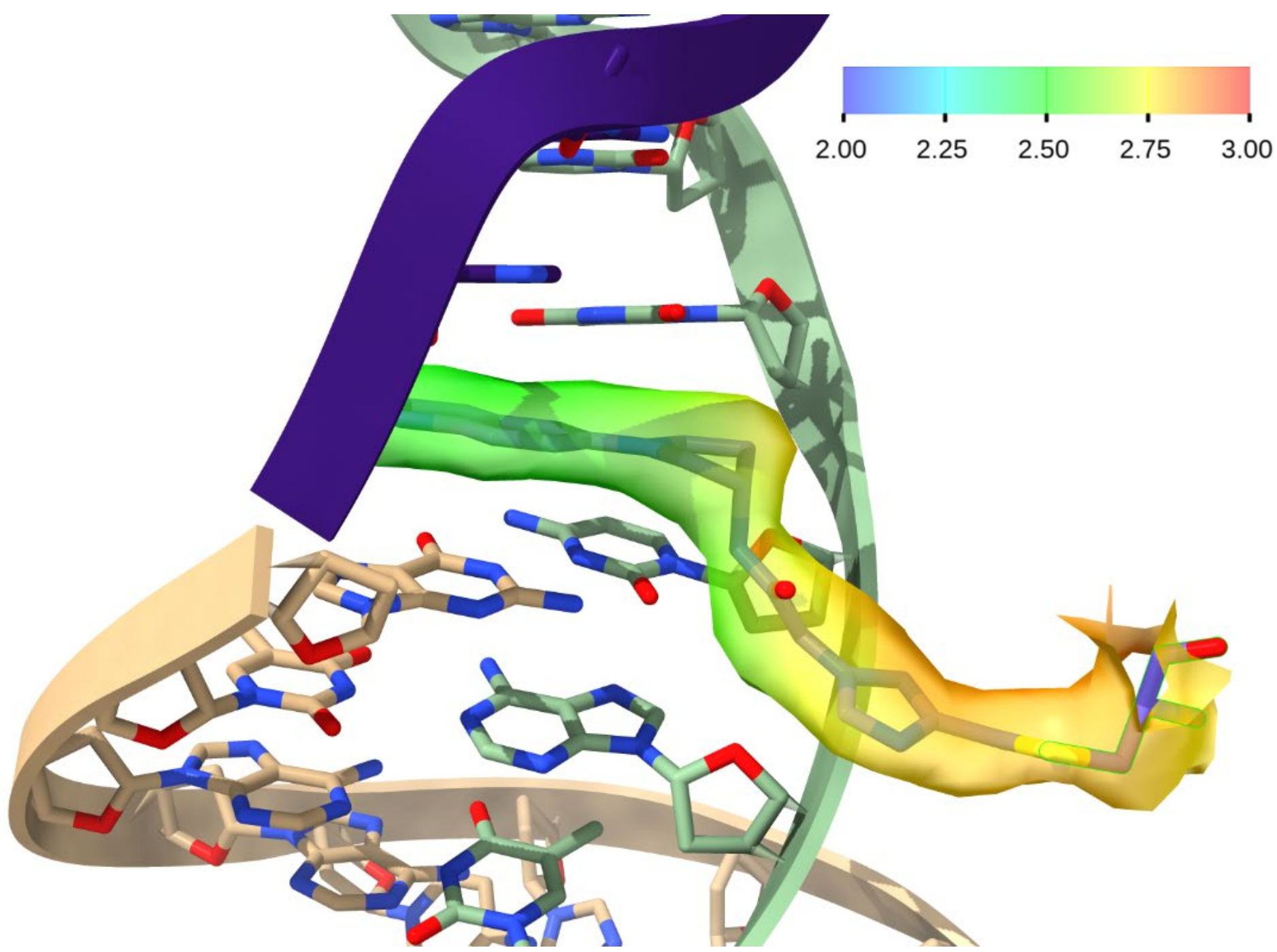

Fig S4. IID432 binds to DNA cleavage site of *T. cruzi* TopoII. (**A**) Representative section around IID432 binding site shows experimental EM map. (**B**). The EM density unambiguously reveals the binding mode of IID432 and shows continuous EM density with cysteine C477 colored by local resolution supporting a covalent binding mode.

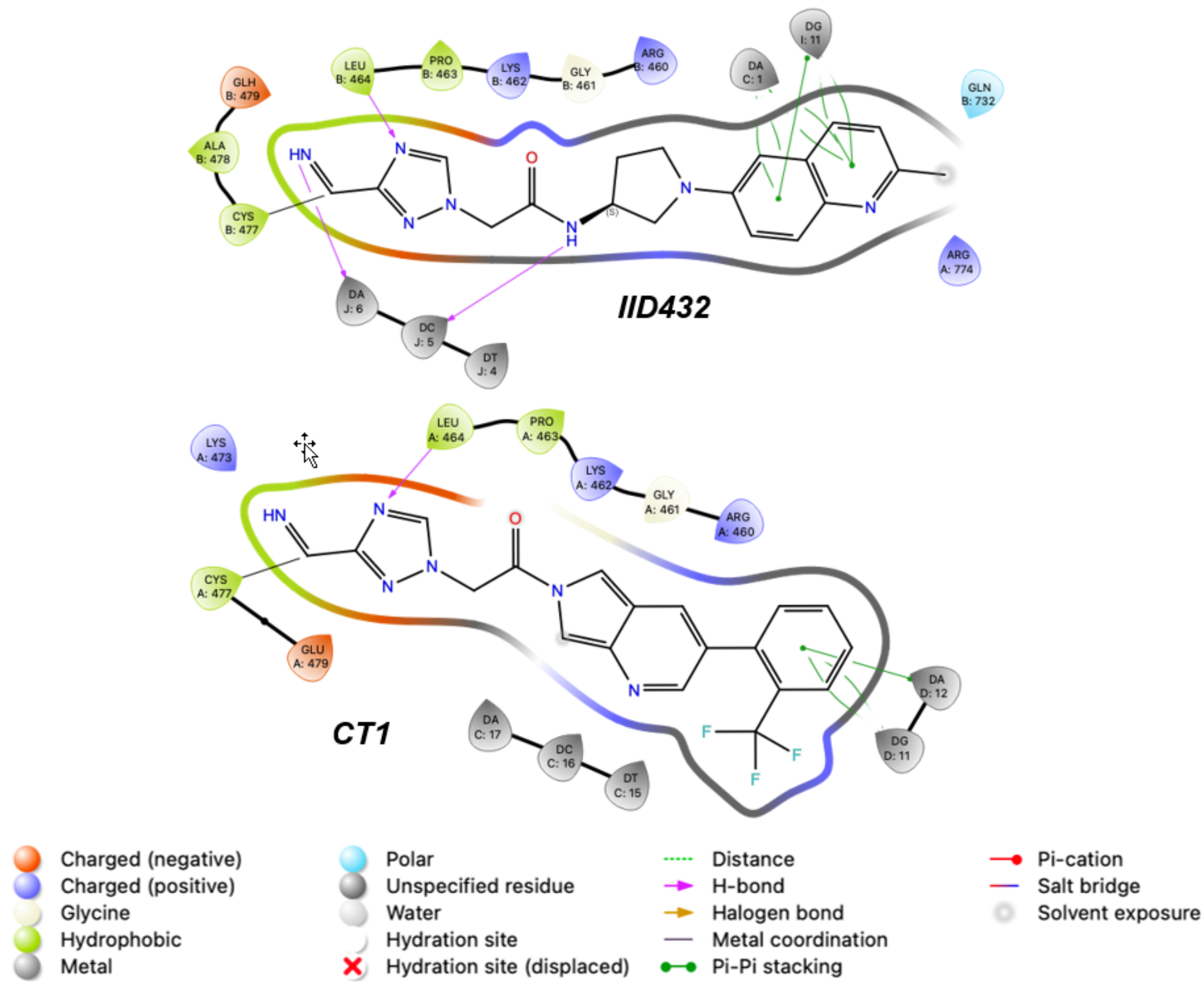

Fig. S5. Ligand interaction diagrams for CT1 and IID432. Ligand interaction after undergoing a protein preparation using default settings in Maesto 2025-2. Both ligands share a covalent bond with Cys477, while IID432 forms additional π–π stacking interactions with DNA bases and a hydrogen bond between the linker NH and the DNA phosphate. Hydrogen bonds are shown as purple lines, π–π interactions as green lines, and the covalent bond with Cys477 as a black line. Molecular renderings and interaction diagrams rendered in Maestro 2025-2.

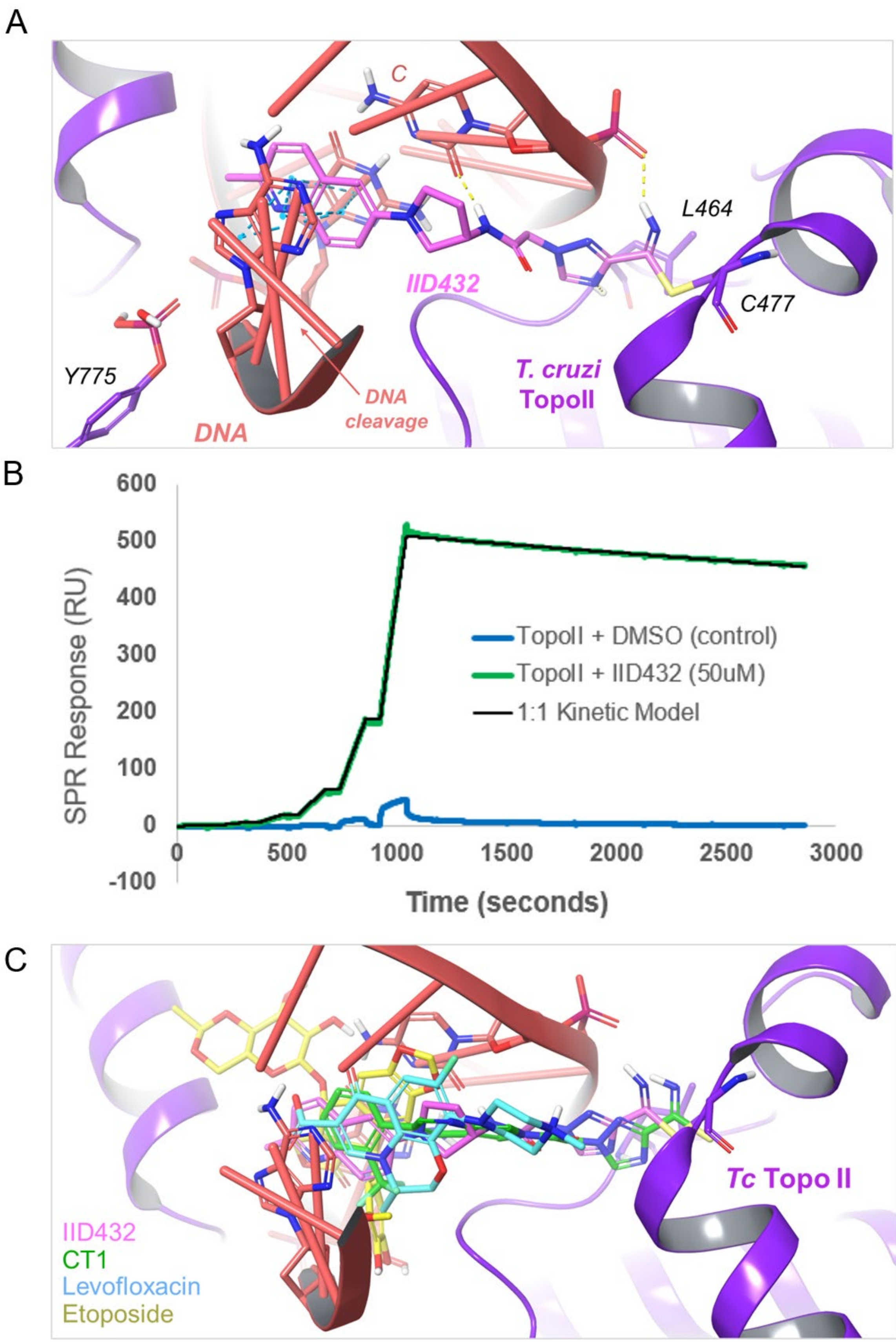
A
C
L464
IID432
C477
Y775
T. cruzi
TopoII
DNA cleavage
DNA
B
600
500
400
300
200
100
0
-100
SPR Response (RU)
0
500
1000
1500
2000
2500
3000
Time (seconds)
TopoII + DMSO (control)
TopoII + IID432 (50uM)
1:1 Kinetic Model
C
Tc Topo II
IID432
CT1
Levofloxacin
Etoposide

Fig S6. Cryo-EM structure and biophysical analysis of IID432 bound to Tc TopoII and dsDNA. (**A**) The quinoline sidechain of IID432 wedges between two nucleotides, getting closer to the catalytic Tyr775 of Tc TopoII. IID432 (pink) bound to Tc TopoII (purple) and DNA (red) complex. (**B**) Representative surface plasmon resonance (SPR) sensorgram showing the association of truncated Tc TopoII (2-1182) with immobilized dsDNA in the presence of IID432 (green) or DMSO control (blue). The 1:1 model of the response is shown in black. Minimal binding occurs in the absence of IID432. The K_D for the interaction of TopoII with dsDNA in the presence of IID432 is 1.7 nM with an dissociation rate (k_d) of 6.1E-5/s and an association rate (k_a) of 36,000/M*sec. (**C**) IID432 (pink) binds to the *T. cruzi* TopoII (purple) DNA cleavage site and occupies a similar site as CT1 (green, PDB 8GCC) and etoposide in human TOP2A (yellow, PDB 5GWK) and the fluoroquinolone levofloxacin in *S. pneumoniae* (blue, PDB 4Z2D). The cyanotriazole group of IID432 and CT1 forms a covalent bond with C477.

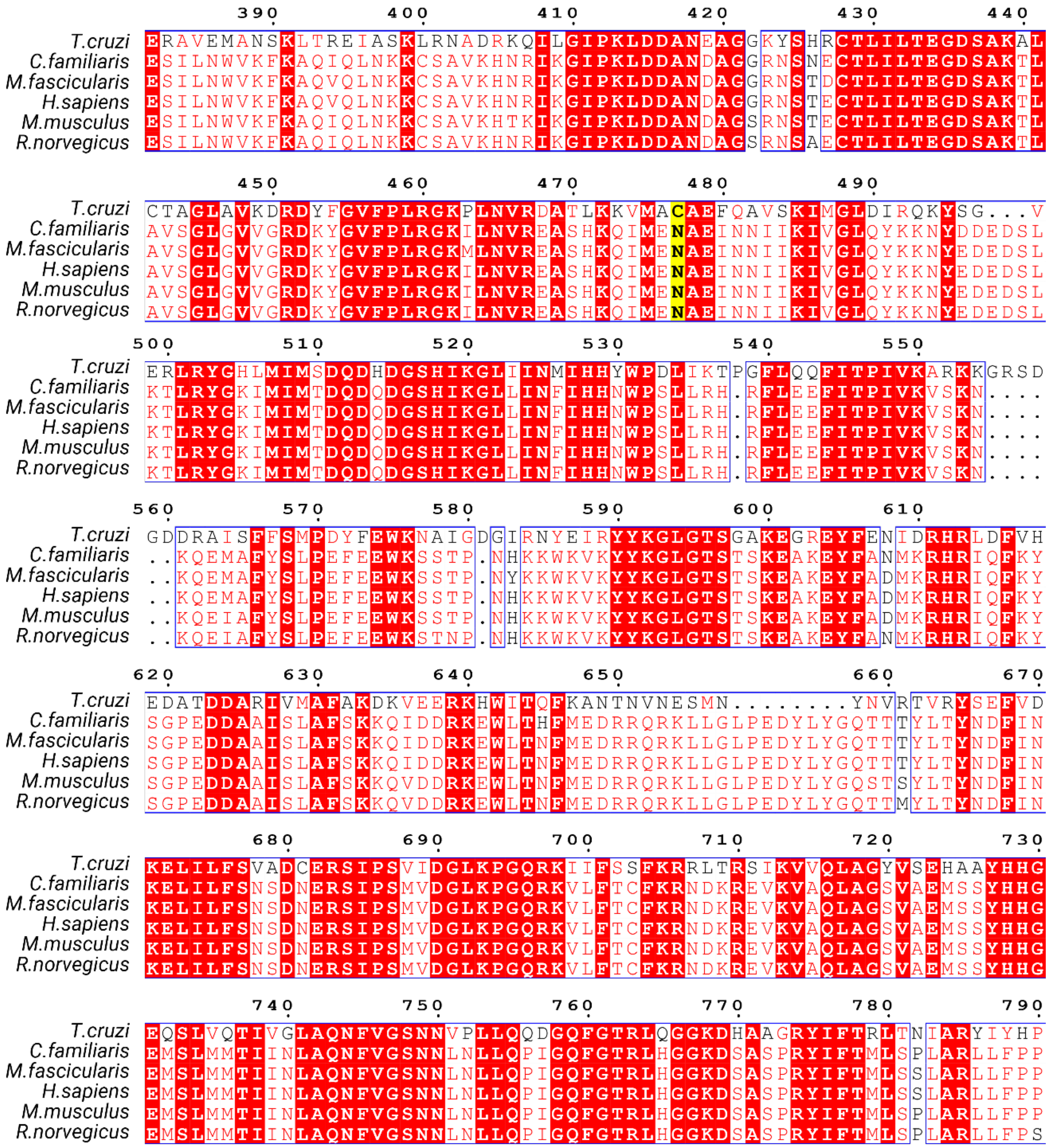

Fig. S7. Multiple sequence alignment of DNA binding region of topoisomerases II sequences. For the sequence alignment, sequences with the following Uniprot identifiers were used: *T. cruzi* (Q4DE53), *M. musculus* (Q01320), *R. norvegicus* (P41516), *C. familiaris* (A0A8I3N3P1), *M. fascicularis* (A0A2K5TYT1), *H. sapiens* (P11388). Sequences were aligned using Clustal Omega and visualized using ESPript. Note the cysteine 477 in *T. cruzi* is asparagine in other mammalian TOP2A.

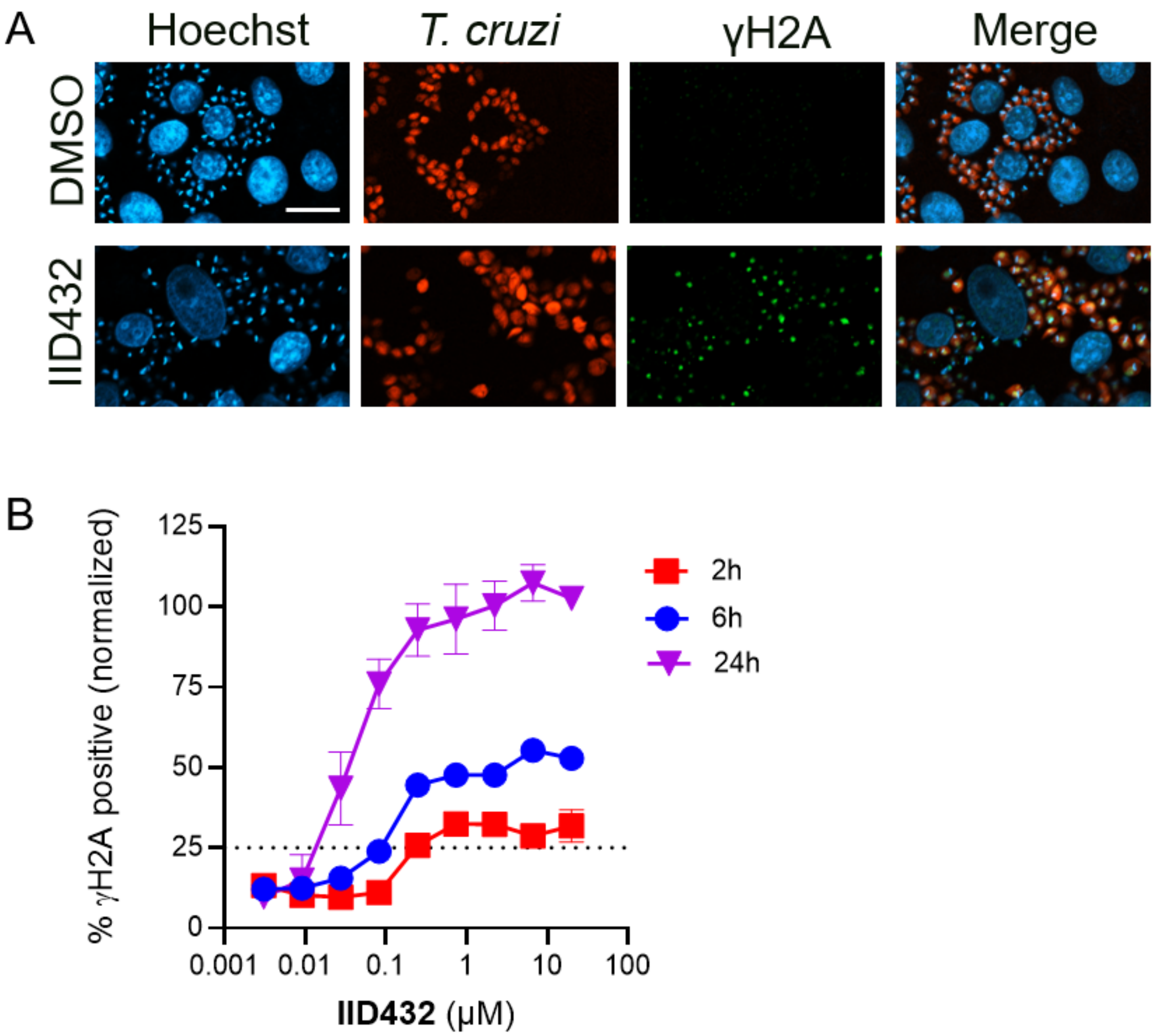

Fig. S8. Kinetics of parasite DNA damage response to IID432 treatment. Vero cells were infected with *T. cruzi* parasites for approximately 48 hours and treated with DMSO or different concentrations of IID432 for either 2, 6 or 24 hours. Infected cells were then fixed, washed and stained for microscopy. (**A**) Representative images of Hoeschst-stained nuclei (blue), intracellular *T. cruzi* expressing tdTomato (red), γH2A induction (green) at 24 hours post-infection are shown. (**B**) Quantification of time and concentration dependent DNA damage response by measuring γH2A induction in *T. cruzi* parasite incubated with 2, 6, or 24 h treatment. Data are normalized to DMSO (neutral control) and CT5 (active control). Data shown are mean and SD of 2 replicates.

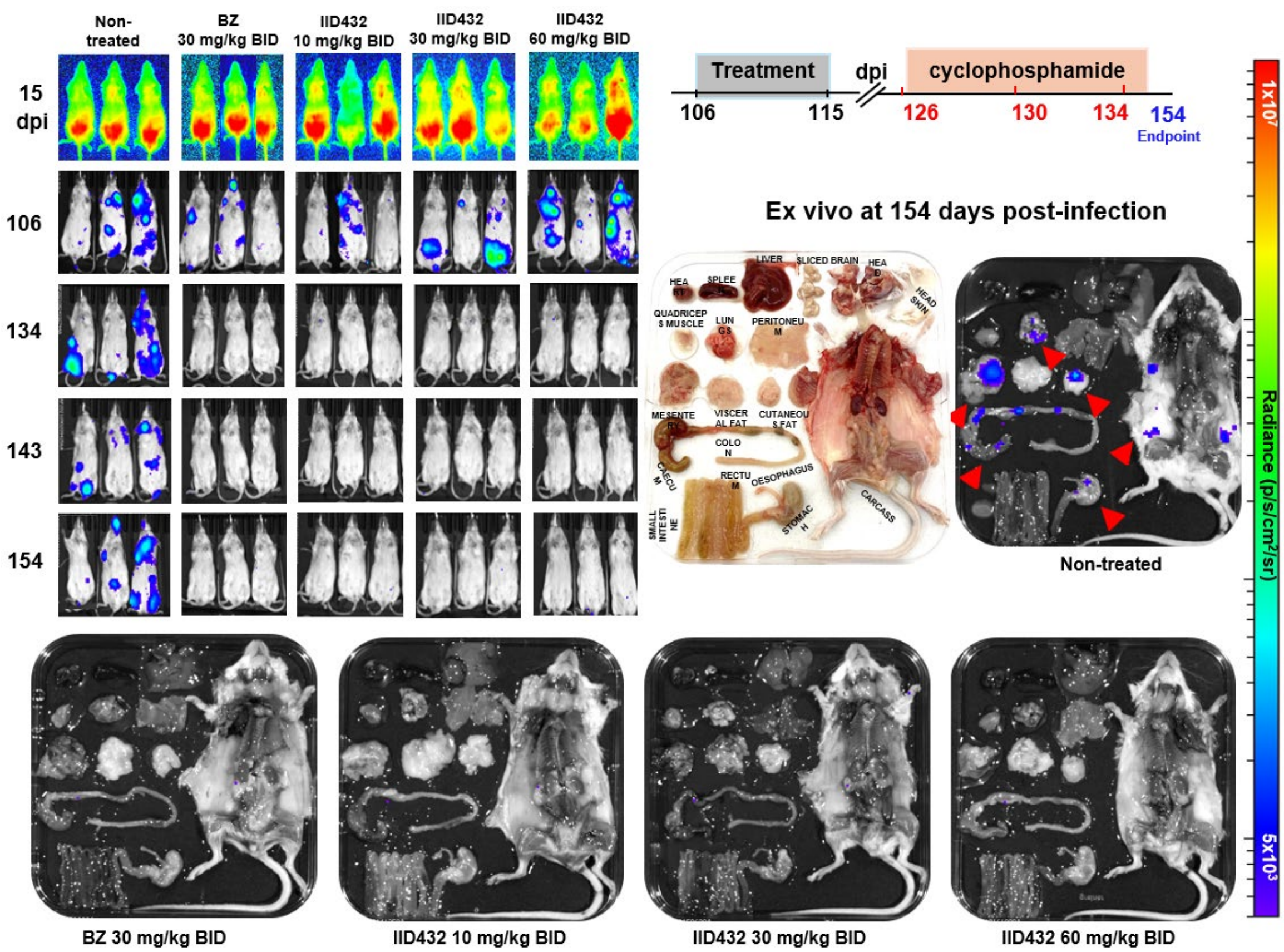
Non-treated
BZ 30 mg/kg BID
IID432 10 mg/kg BID
IID432 30 mg/kg BID
IID432 60 mg/kg BID
15 dpi
106
134
143
154
Treatment
dpi
cyclophosphamide
106
115
126
130
134
154
Endpoint
Ex vivo at 154 days post-infection
LIVER
SLICED BRAIN
SPLEE
QUADRICEPS MUSCLE
PERITONEUM
VISCERAL FAT
CUTANEOUS FAT
OESOPHAGUS
STOMACH
CARCASS
Non-treated
Radiance (p/s/cm²/sr)
1x10⁷
5x10³
BZ 30 mg/kg BID
IID432 10 mg/kg BID
IID432 30 mg/kg BID
IID432 60 mg/kg BID

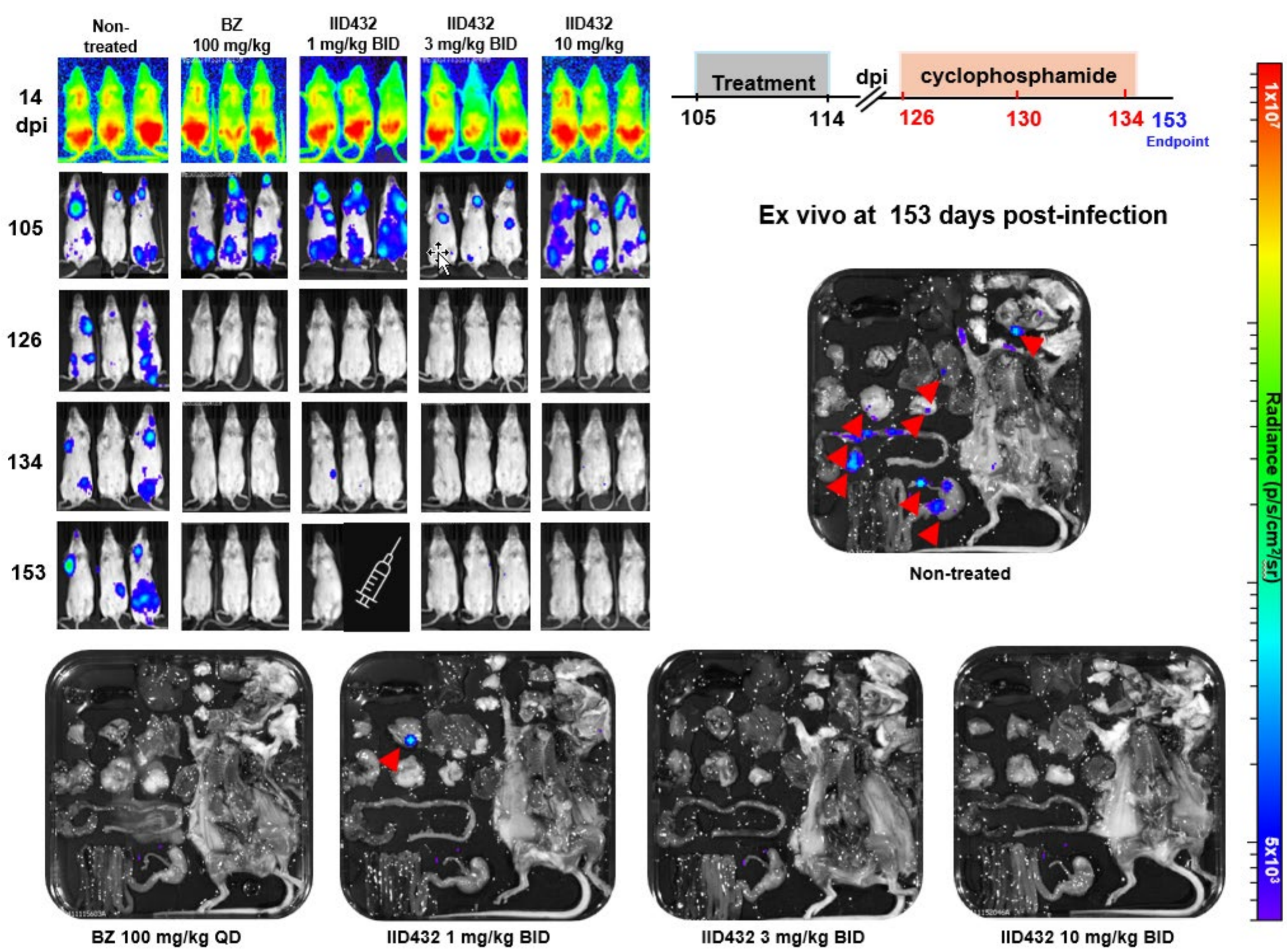
Non-treated
BZ 100 mg/kg
IID432 1 mg/kg BID
IID432 3 mg/kg BID
IID432 10 mg/kg
14 dpi
105
126
134
153
Treatment
dpi
cyclophosphamide
105
114
126
130
134
153
Endpoint
Ex vivo at 153 days post-infection
Non-treated
Radiance (p/s/cm²/sr)
1x10⁷
5x10³
BZ 100 mg/kg QD
IID432 1 mg/kg BID
IID432 3 mg/kg BID
IID432 10 mg/kg BID

Fig. S9. In vivo and ex vivo assessment of non-treated, benznidazole- and IID432-treated mice during chronic stage *Trypanosoma cruzi* infection. BALB/c mice were longitudinally followed, and representative in vivo bioluminescence images were acquired at multiple time points during infection, as shown. At the chronic stage of infection, mice were treated orally for 10 days with vehicle (non-treated), benznidazole (BZ; 30 mg/kg BID or 100 mg/kg QD), or IID432 at 1, 3, 10, 30 or 60 mg/kg BID, beginning at 105-106 days post-infection (dpi). QD: once daily. BID: twice daily. The left panel shows representative ventral in vivo bioluminescence images from three mice per group at the indicated time points. On days 126, 130 and 134, mice bioluminescent negative by in vivo imaging were immunosuppressed with cyclophosphamide (200 mg/kg intraperitoneally) to assess parasite recrudescence. Mice #2 and #3 IID432 treated at 1 mg/kg BID were bioluminescence positive and were humanely killed on day 143 (syringe icon) prior to the experimental endpoint. At the endpoint (153-154 dpi), organs from one representative mouse per group that remained bioluminescence negative by in vivo imaging were analysed by ex vivo imaging. The organ atlas identifies the tissues and organs examined and their anatomical positions. Ex vivo imaging revealed residual bioluminescent foci in non-treated mice and in mice treated with IID432 at 1 mg/kg BID, whereas no detectable bioluminescence was observed in the representative mice treated IID432 at all doses ≥3 mg/kg BID. Red arrows indicate sites of parasite persistence. Heat maps are shown on a log10 scale, with bioluminescence intensity ranging from low (blue) to high (red).

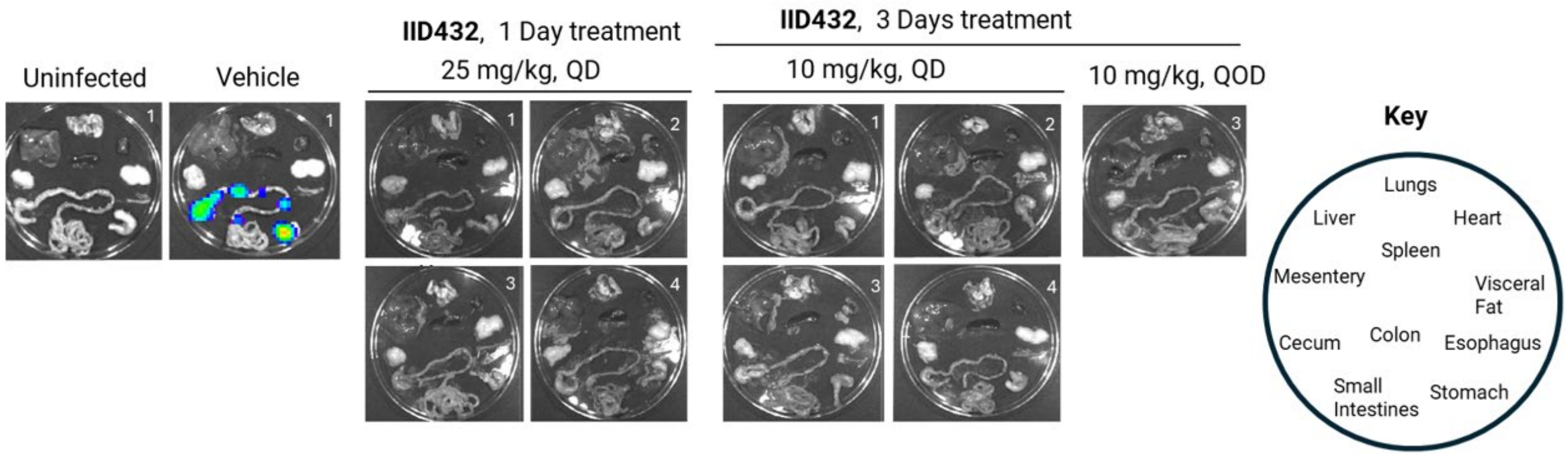

Fig. S10. Ex vivo imaging of mice treated with varying doses of IID432 at the end of the study. Organs were collected at the end of the study (day 136) and imaged ex vivo using IVIS system. Representative images are shown for uninfected mice, infected mice treated with vehicle, and infected mice treated with IID432 under different dosing regimens. Treatment groups include: 25 mg/kg single dose (1-day treatment), 10 mg/kg once daily (QD) for 3 days, and 10 mg/kg every other day (QOD). Four mice are shown for the 25 mg/kg and 10 mg/kg QD groups, and one mouse for the 10 mg/kg QOD group. Labelled numbers correspond to individual mice from which the organs were collected. The key indicates organ identity and their spatial arrangement on the Petri dish. QD, once daily; QOD, once every other day.

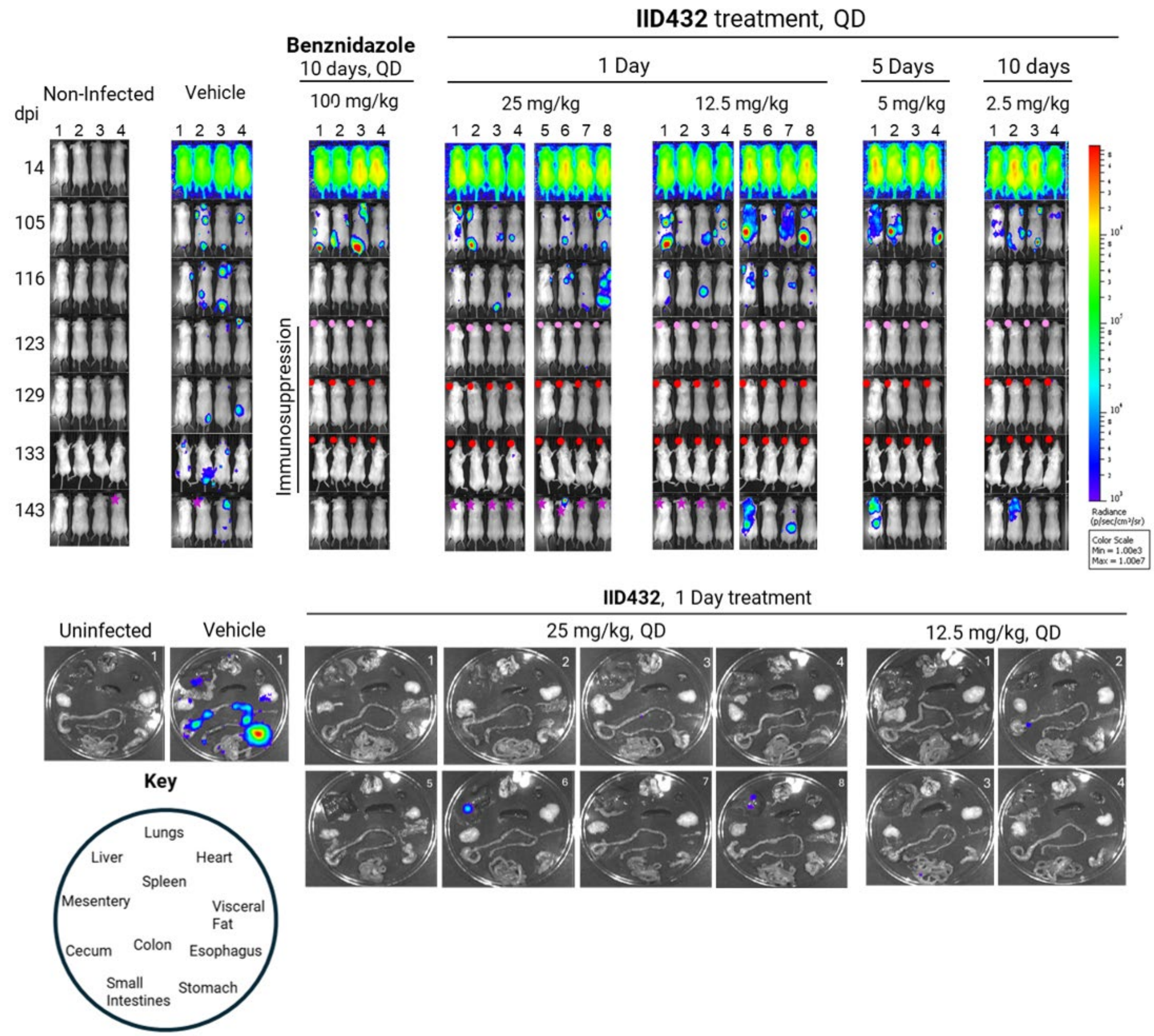

Fig. S11. **Dose fractionation efficacy study of IID432 in a murine model of chronic Chagas disease for determination of PK/PD analysis.** BALB/c mice chronically infected with *T. cruzi* (chronic stage) were treated with IID432 using different dosing regimens: single doses of 25 or 12.5 mg/kg (n = 8), 5 mg/kg once daily (QD) for 5 days (n = 4), or 2.5 mg/kg QD for 10 days, starting at ~day 100 post-infection. Mice were imaged longitudinally using an IVIS system, and bioluminescence was quantified at the experimental endpoint (day 143). Dorsal images are shown at selected time points. Untreated controls were imaged at matched time points and were not immunosuppressed. To assess parasite recrudescence, treated mice were immunosuppressed with cyclophosphamide on days 123, 129, and 133 (see Methods). Pink dots indicate the first round of immunosuppression, red dots the second and third rounds, and purple stars indicate mice subjected to ex vivo imaging. Individual mice were tracked throughout the study. Ex vivo imaging was performed at the endpoint (day 143) on organs collected from mice in the 25 mg/kg (n = 4) and 12.5 mg/kg (n = 4) groups. In addition, ex vivo imaging of organs from uninfected mice and infected mice treated with vehicle was included as controls. Labelled numbers correspond to individual mice, and the key indicates organ identity and position on the petridish.

A

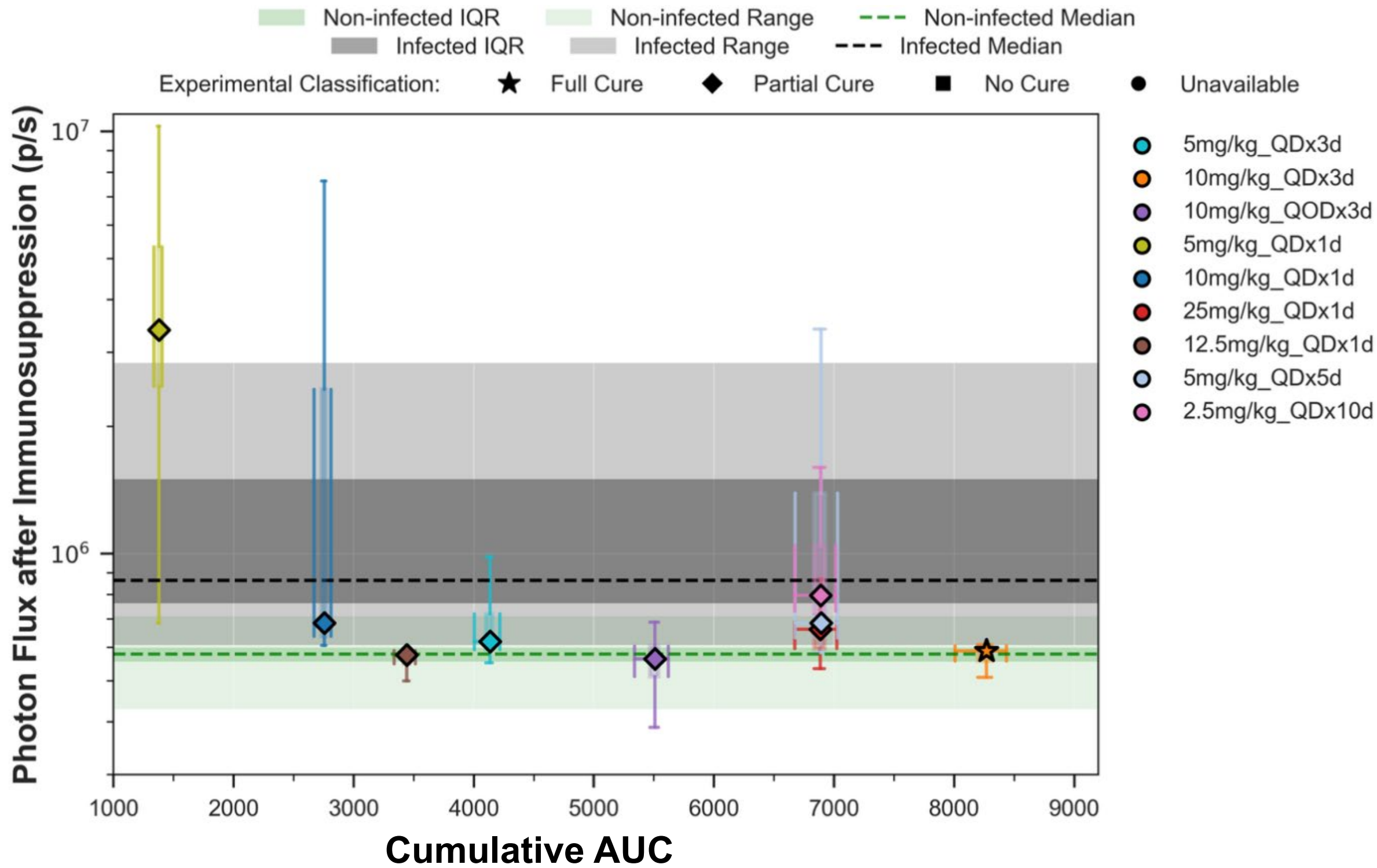

B

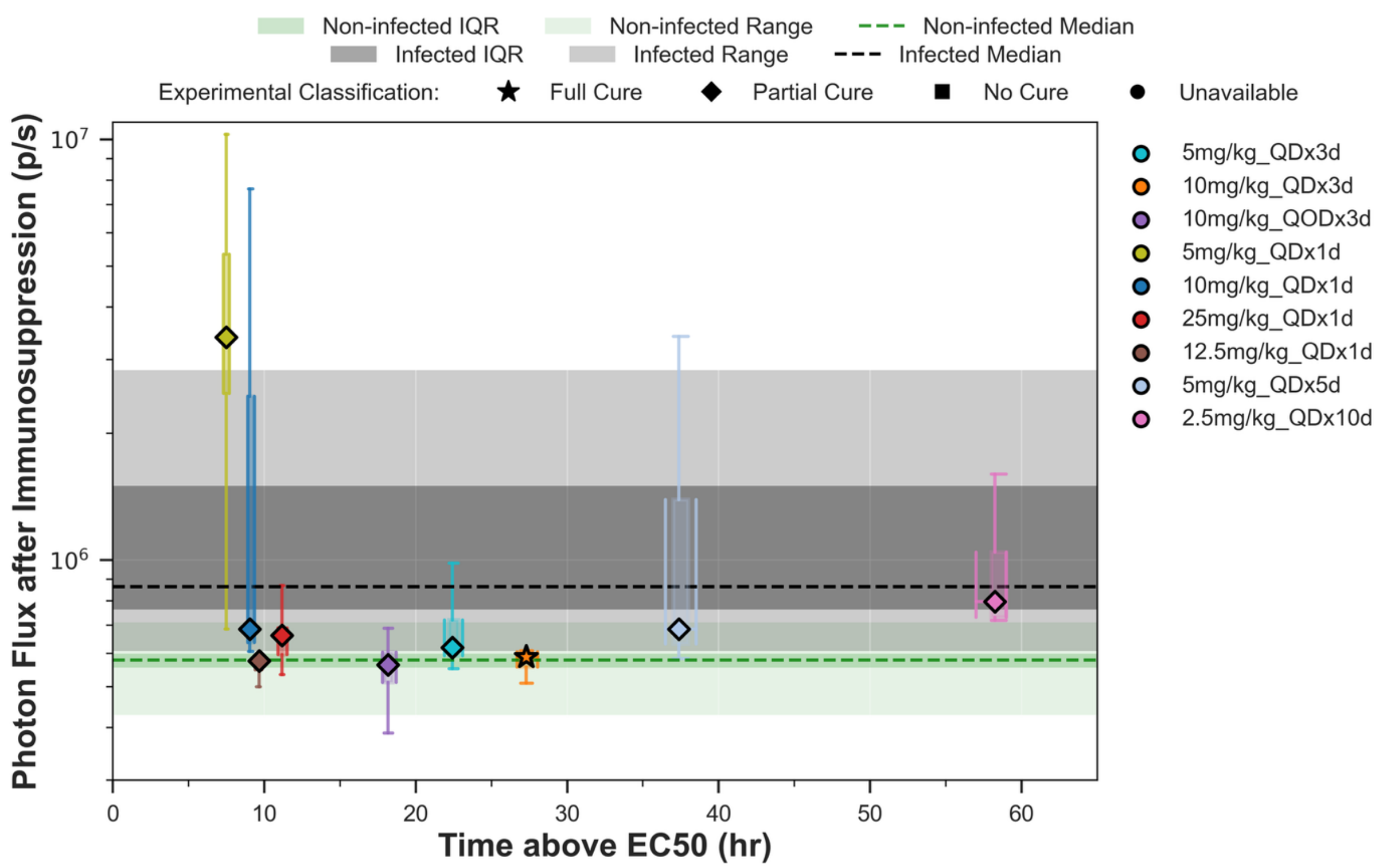

Fig. S12. Population PK/PD relationship for IID432 in mice efficacy studies. (**A**) Cumulative AUC derived from population PK modelling and their respective median photon flux after

immunosuppression in shown for multiple regimens. At the population level there is good match between the quantitative and the qualitative classification of full cure. The cumulative AUC threshold was defined as the minimal value such that the range of cumulative AUC values (when accounting for individual PK variability) for all dose groups above this threshold achieved a full cure, corresponding with the left end of the horizontal range of the 10 mg/kg QD x 3 days treatment (a value of ~8000 ng/ml*h). (**B**) Median time above EC50 derived from population PK modelling and their respective median photon flux after immunosuppression is shown for multiple dosing regimens. The trend in the population PK/PD plot is nonmonotonic – the most efficacious treatment is not the one with the longest time above EC_{50} – which suggests time above EC_{50} is not the PK driver of efficacy for IID432.

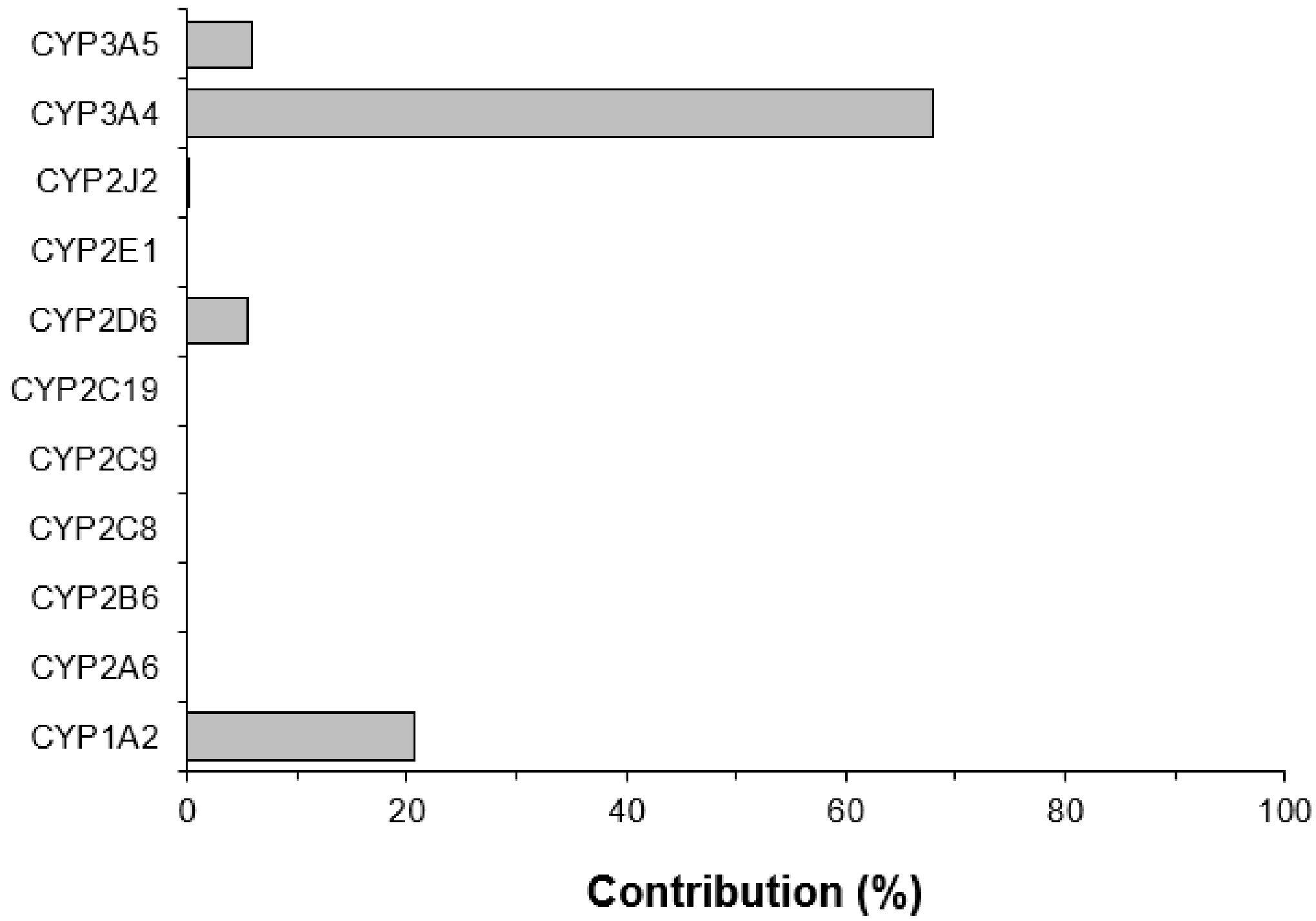

Fig. S13. Percentage contribution of individual CYP isoenzymes to the total CYP mediated oxidative metabolism of IID432 using human liver microsomes.

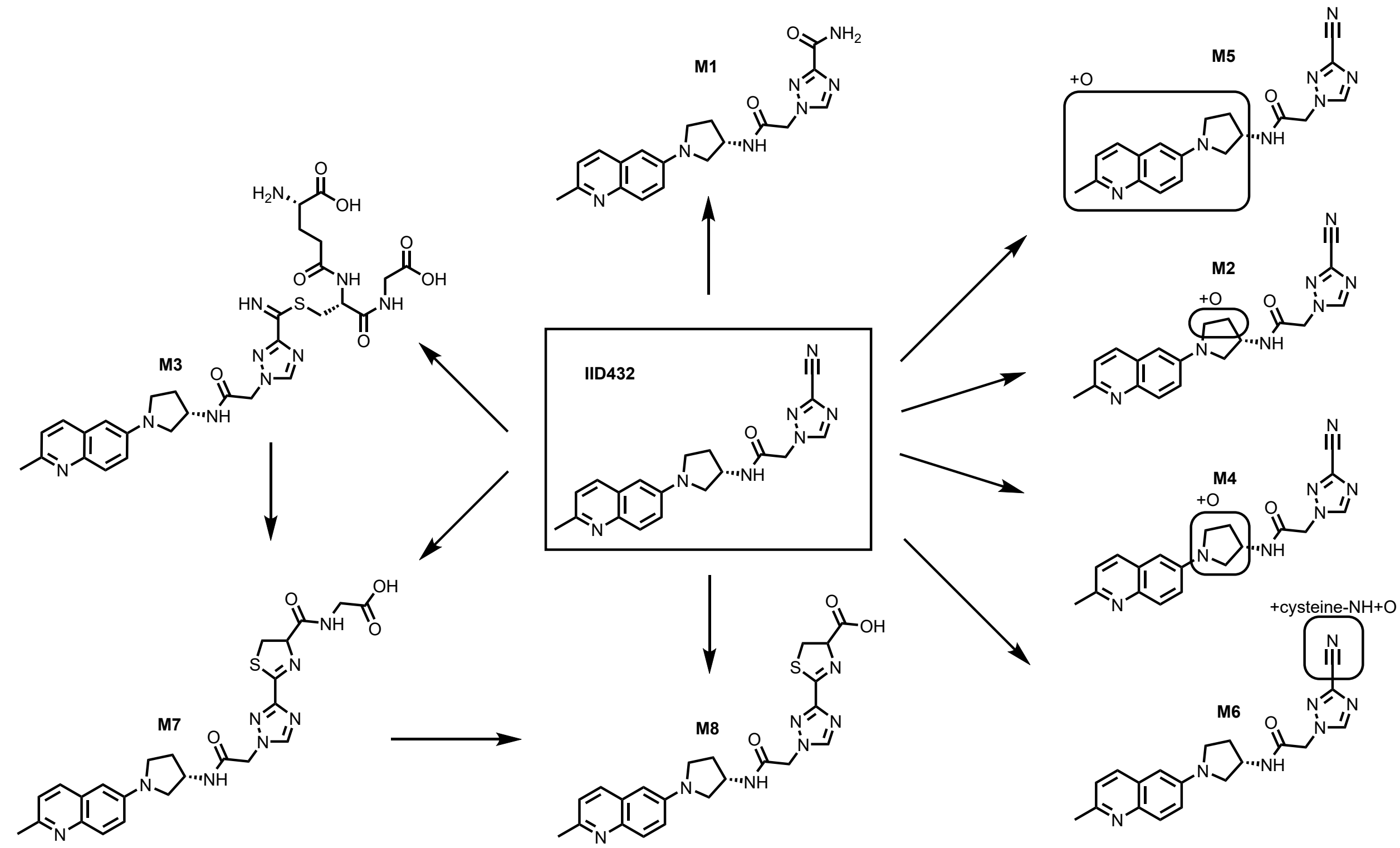

Fig. S14. Proposed metabolic pathway of IID432 in preclinical species and human liver S9 incubations.

Tables

Table S1. Cryo-EM data collection, refinement, and validation statistics.

	**TopoII DNA binding domain bound to dsDNA and IID432**	
**Data collection and processing**		
EMDB ID	EMD-59152	
Microscope	ThermoFisher Titan Krios G2	
Magnification (nominal)	75000	
Magnification (calibrated at detector)	165680	
Voltage (kV)	300	
Total electron exposure (e-/Å2)	30	
EER fractionation	30	
Pixel size (Å)	0.845	
Automation software (EPU, SerialEM, Leginon)	EPU 3.0	
# of particles in final map	398902	
Resolution at 0.143 FSC (masked/unmasked)	2.8/3.4	
Resolution at 0.5 FSC (masked/unmasked)	3.63/3.71	
Resolution half-bit FSC (masked/unmasked)	2.80/2.81	
cFAR	0.47	
Map sharpening B factor (Å2)	113.9	
	**30° tilted data**	**untilted data**
Detector	ThermoFisher Falcon 4	ThermoFisher Falcon 4
Defocus range (µm)	1.4-1.8	0.6-2.0
Total electron exposure (or fluence, e-/Å2)	30	
EER fractionation	30	
# Micrographs used	5801	5327
Total # of extracted particles	1674822	3376090
**Structure**		
PDB-ID	pdb_000032to	
**Model composition**		
Non-hydrogen atom	11388	
protein residues	1382	
DNA	48	
Ligands	2	
Atomic modeling refinement package(s)	Phenix 2.1-6048	
CCvolume/CCmask	0.81/0.83	
**B factors**		
protein residues	15.88/157.87/72.26	
DNA	20.31/98.19/46.27	
Ligands	27.69/76.01/42.81	
**RMSD**		

Length (Å)	0.002
Angles (°)	0.524
Molprobity score	1.97
All-atom clashscore	6.62
Rotamer outliers (%)	2.52
**Ramachandran percentiles (%)**	
Favored	95.62
Allowed	4.38
Outliers	0.00
CaBLAM outliers (%)	3.68
EMRinger score	1.8

CC, cross correlation; cFAR, conical FSC Area Ratio; DNA, deoxyribonucleic acid; EER, electron-event representation (file format); EMDB, Electron Microscopy Data Bank; FSC, Fourier shell correlation; PDB, Protein Data Bank

Table S2. Efficacy of IID432 for the treatment of chronic infection with *T. cruzi* in BALB/c mice conducted at London School of Hygiene and Tropical Medicine.

Compound	Dose (mg/kg)	Regimen	Number of mice negative by BLI/ number of treated mice (Cure rate)	
			End of study#	Ex vivo imaging#
IID432	1	BID, 10 days	0/5 (0%)	0/5 (0%)
	3	BID, 10 days	4/4* (100%)	4/4* (100%)
	10	BID, 10 days	5/5 (100%)	5/5 (100%)
	10	QD, 10 days	5/5 (100%)	5/5 (100%)
	30	BID, 10 days	5/5 (100%)	5/5 (100%)
	60	BID, 10 days	5/5 (100%)	5/5 (100%)
Benznidazole	30	BID, 10 days	2/3 (67%)	2/3 (66.67%)
	100	QD, 10 days	3/3 (100%)	3/3 (100%)

BLI, bioluminescence imaging; BID, dosed two times a day; QD, dosed once a day.

#End of study: Whole body imaging of all mice, after 3 rounds of immunosuppression and waiting period of 10 days. Ex vivo imaging: Organs from all mice were excised and assessed for presence of parasites using bioluminescence imaging.

*One mouse was humanely euthanized before the end of cyclophosphamide treatment and was excluded from the final analysis.

Table S3. Efficacy of IID432 for the treatment of chronic infection with *T. cruzi* in BALB/c mice carried out at Novartis facility.

Dose (mg.kg^{-1})	Regimen	Total dose	Number of mice below LLOQ of BLI/number of treated mice (%)	
			End of study#	Ex vivo imaging#
Experiment 1				
5	QD, 3 days	15	3/4 (75%)	NA
10	QD, 3 days	30	4/4 (100%)	4/4 (100%)
10	QOD, 3 days	20	3/4 (75%)	1/1*
5	QD, 1 day	5	1/4 (25%)	NA
10	QD, 1 day	10	3/4 (75%)	NA
25	QD, 1 day	25	4/4 (75%)	4/4 (100%)
Experiment 2				
2.5	QD, 10 days	25	1/4 (25%)	NA
5	QD, 5 days	25	3/4 (75%)	NA
12.5	QD, 1 day	12.5	4/8 (50%)	2/4*
25	QD, 1 day	25	5/8 (62.5%)	5/8 (62.5%)

LLOQ, lower limit of quantification; QD, once daily; QOD, once every other day; NA, not assessed; #End of study: Whole body imaging of all mice, after 3 rounds of immunosuppression and waiting period of 10 days. Ex vivo imaging: Organs from all mice were excised and assessed for presence of parasites using bioluminescence imaging. *, only mice which had below LLOQ of BLI were assessed ex vivo.

Table S4. Pharmacokinetic analysis of mice blood samples from Chronic Chagas efficacy model. Mean total Cmax, and AUC 0-7h of IID432 following oral administration in BALB/c mice for the treatment of infection with *T. cruzi* in a chronic mice model.

Dose (mg/kg)	Dosing Regimen	PK sample collection (day)	Number of animals	Tmax (h) [range]	Cmax (ng/mL)	AUC0-7h (ng*hr/mL)
Experiment 1						
5	QD, 3 days	3	4	1.00 [1.0-1.00]	331.0 ± 249.7	784.2 ± 552.9
10	QD, 3 days	3	4	1.00 [1.0-1.00]	639.7 ± 175.6	1698.6 ± 509.6
25	QD	1	4	1.00 [1.0-1.00]	2829.8 ± 162.6	7155.72± 422.8
Experiment 2						
25	QD	1	8	0.5 [0.5-0.5]	1850.4 ± 578.2	5457.1 ± 1651.6
12.5	QD	1	8	0.5 [0.5-0.5]	972.2 ± 211.4	2255.1 ± 348.4
5	QD, 5 days	5	4	0.5 [0.5-0.5]	358.5 ± 37.6	842.1 ± 126.1
2.5	QD, 10 days	10	4	0.5 [0.5-0.5]	90.4 ± 16.1	221.2 ± 23.5

Blood from mice infected *T. cruzi* treated with above doses of IID432 were collected at 3 different time points. The drug concentration at each time was measured using LC/MS method as described in methods section. PK parameters were determined using WinNonLinTM version 8.3 software.

PK parameters are presented as mean ± standard deviations. QD: once a day. Tmax: time of peak concentration; Cmax: maximum blood concentration; AUC: area under the curve (t = 0 to 7hours)

Table S5. Population PK/PD analysis of chronic mice efficacy experiment.

Dose mg/kg	Dosing Regimen	Cumulative AUClast (ng*h/mL)	Cmax (ng/mL)	Cumulative T>TcEC$_{50}$ (h)	*Cured /total (%)	Classification
**5**	QD, 1 day	1377 [1145–1662]	1431 [680–3941]	7.5 [5.8–9.5]	1/4 (25%)	Partial cure
**10**	QD, 1 day	2753 [2289–3324]	2863 [1360–7881]	9.1 [7.0–12.3]	3/4 (75%)	Partial cure
**12.5**	QD, 1 day	3442 [2861–4157]	3578 [1700–9852]	9.7 [7.4–13.2]	4/8 (50%)	Partial cure
**25**	QD, 1 day	6887 [5725–8313]	7157 [3401–19703]	11.2 [8.4–16.4]	9/12 (75%)	Partial cure
**10**	QOD, 3 days	5507 [4577–6648]	2863 [1360–7881]	18.15 [13.9–24.700]	3/4 (75%)	Partial cure
**5**	QD, 3 days	4132 [3436–4984]	1431 [680–3941]	22.4 [17.3–28.4]	3/4 (75%)	Partial cure
**10**	QD, 3 days	8263 [6877–9972]	2863 [1360–7882]	27.3 [20.9–37.0]	4/4 (100%)	Full cure
**5**	QD, 5 days	6890 [5729–8309]	143 [680–3941]	37.4 [28.8–47.4]	3/4 (75%)	Partial cure
**2.5**	QD, 10 days	6894 [5729–8321]	716 [340–1,971]	58.3 [43.5–72.0]	1/4 (25%)	Partial cure

Note: PK parameters were derived from population PK modeling as described in materials and methods section. Population parameters were used to perform 10 replicate in-silico trials with 100 virtual mice each, selected according to the fitted statistical distributions of the parameters. For each replicate trial, the median, 5th percentile and 95th percentile across virtual mice were calculated, and the median of these values across replicates are reported in this summary table as "median [5th percentile – 95th percentile]".

*Data from both experiment 1 and 2 were pooled to determine the ratio of total number of mice cured at the end of the study mice versus total mice in the study.

QD: once daily, QOD: Once every other day. Cmax: maximum blood concentration; T>TcEC50: time above *T. cruzi* EC$_{50}$; AUClast: area under the curve (t = 0 to infinite)

Table S6. Physicochemical and in vitro ADME properties of IID432.

**Properties**	**IID432**
Molecular Weight (Da)	361.4
Solubility (pH 6.8, 4.0, FaSSIF) (μM)	402, >750, >750
Lipophilicity (logP, logD)	2.2, 2.2
MDCK Low Efflux (PappA-B, x10-6, cm s-1)	12.4
MDCK MDR1(Papp A-B/ B-A (x10-6, cm s-1)/ ratio	1.8 / 26 / 14.9
***In vitro* stability (M/R/D/C/H)**	
Microsomal CLint [μl/min/mg]	<25 / 45.3 / 40.9 / 24.5 / <25
Microsomal ER (%)	32 / 58 / 70 / 26 / 55
Hepatocytes [μl/min/million cells]	- / 17.8 / <4 / <4 / <4
Hepatocytes ER (%)	- / 59 / 41 / 18 / 33
Intestinal S9 T1/2 (min)	>205 / >240 / >240 / - / 194
Plasma T1/2 (min)	>240 / >240 / >240 / - / >240
**% Plasma protein binding** M/R/D/C/H	93.1/ 93.1/ 78.5 / 76.7 / 92.9
Blood: Plasma ratio M/R/D/C/H	0.852/0.776/1.02/0.960/0.665
**CYP P450 isoforms inhibition (IC_{50} μM)**	
Reversible 3A4 / 2D6 /2C9	>100 / >100 / >100
Time dependent inhibition 3A4	Negative
CYP3A4 / 1A2 / 2B6 hepatocyte induction assay	Non-inducer

Da: Dalton; FaSSIF: Fasted state simulated intestinal fluid; logP: logarithm of partition co-efficient; logD: logarithm of distribution co-efficient; MDCK: Madin-Darby canine kidney; MDCK MDR1: MDCK with multi drug resistance mutation; Papp: apparent permeability coefficient; Clint: Clearance; ER: Extraction ratio; M: mouse; R: rat; D: dog; C: cyno; H: human

Table S7. Proposed metabolic pathway of IID432 in animal and human liver S9 incubations.

Metabolite	Metabolic reaction	Species			
		Rat	Dog	NHP	Human
M1[a]	Biotransformation of nitrile to amide	x	x	x	x
M2	Mono-oxidation in pyrrolidine ring	x	x	x	t
M3[a]	Glutathione addition to nitrile	x	x	x	x
M4	Mono-oxidation in pyrrolidine ring	x	x	x	t
M5	Mono-oxidation in pyrrolidine/methylquinoline moiety	x	x	x	t
M6	Cysteine addition to nitrile and oxidative metabolism (-NH+O)	x	x	x	x
M7	Cysteine-glycine addition to nitrile and rearrangement to form a thiazoline	x	x	xx	x
M8	Cysteine addition to nitrile and rearrangement to form a thiazoline	x	x	x	xx
IID432		xxx	xxx	xxx	xxx

xxx = detected at > 75% of the relative abundance of the largest peak

xx = detected at 25-75% of the relative abundance of the largest peak

x = detected at 1-25% of the relative abundance of the largest peak

t = detected at 0-1% of the relative abundance of the largest peak

a: Formed at similar levels in incubations without liver S9, suggesting these metabolites may result from non-enzymatic processes

NHP: non-human primate

Table S8. Summary of pharmacokinetic parameters of IID432 in blood or plasma after single oral dose and intravenous dose.

Parameters	Mouse	Rat			Dog	Monkey
			Oral pharmacokinetics			
Nominal Dose (mg/kg)	30	30			1	10
Matrix	Blood	Blood			Blood	Plasma
Cmax (ng/mL)	5070 ± 402	3550 ± 956			80.2 ± 14.3	773 ± 650
Tmax (h)	0.5 [0.25-1.0]	1.0 [1.0-1.0]			0.5 [0.5-0.5]	1.0 [1.0-4.0]
AUClast (ng.h/mL)	11000 ± 521	14300 ± 3870			139 ± 30.6	2300 ± 1460
AUCinf (ng.h/mL)	11000 ± 521	14300 ± 3870			142 ± 31.4	2310±1460
%F	88	52			75	43
			Intravenous pharmacokinetics			
Nominal dose (mg/kg)	1	0.3	1	3	0.3	1
Matrix	Blood	Plasma	Plasma	Plasma	Blood	Plasma
AUClast (ng.hr/mL)	418 ± 34.8	255 ± 63.8	1050 ± 159	3600 ± 216	55.3 ± 10.2	531 ± 119
AUCinf (ng.hr/mL)	419 ± 35.3	279 ± 58.9	1080 ± 156	3620 ± 218	57.1 ± 10.8	534 ± 120
CL (ml/min/kg)	40 ± 3.53	19.00 ± 3.40	15.64 ± 2.40	14.17 ± 1.03	89.6 ± 16.8	32.2 ± 6.35
Vss (L/kg)	2.09 ± 0.39	1.06 ± 0.079	0.942 ± 0.201	0.970 ± 0.108	5.68 ± 0.91	2.9 ± 0.23
$T_{1/2}$	0.88	0.736 ± 0.192	0.819 ± 0.116	0.967 ± 0.0317	0.85	1.4 ± 0.27

Cmax: maximum blood concentration; Tmax: time of peak concentration; AUC: area under the curve (*t* = 0 to infinite); F: absolute oral bioavailability; Vss: volume of distribution at steady state; CL: clearance; $T_{1/2}$: elimination half-life.

SI References

1. A. M. C. Canavaci, *et al.*, In vitro and in vivo high-throughput assays for the testing of anti-Trypanosoma cruzi compounds. *PLoS Negl. Trop. Dis.* **4**, e740 (2010).
2. S. Khare, *et al.*, Proteasome inhibition for treatment of leishmaniasis, Chagas disease and sleeping sickness. *Nature* **537**, 229–233 (2016).
3. S. P. S. Rao, *et al.*, Cyanotriazoles are selective topoisomerase II poisons that rapidly cure trypanosome infections. *Science* **380**, 1349–1356 (2023).
4. D. Liebschner, *et al.*, Macromolecular structure determination using X-rays, neutrons and electrons: recent developments in Phenix. *Acta Crystallogr. D Struct. Biol.* **75** (2019).
5. O. S. Smart, *et al.*, Exploiting structure similarity in refinement: automated NCS and target-structure restraints in BUSTER. *Acta Crystallogr. D Biol. Crystallogr.* **68** (2012).
6. F. Long, *et al.*, AceDRG: a stereochemical description generator for ligands. *Acta Crystallogr. D Struct. Biol.* **73**, 112–122 (2017).
7. R. S. Jumani, B. Thomas, S. P. S. Rao, Imaging Assays to Detect DNA Damage in Trypanosome Parasites Using γH2A. *Bio. Protoc.* **14**, e5026 (2024).
8. M. D. Lewis, *et al.*, Bioluminescence imaging of chronic Trypanosoma cruzi infections reveals tissue-specific parasite dynamics and heart disease in the absence of locally persistent infection. *Cell. Microbiol.* **16** (2014).
9. A. F. Francisco, *et al.*, Limited Ability of Posaconazole To Cure both Acute and Chronic Trypanosoma cruzi Infections Revealed by Highly Sensitive In Vivo Imaging. *Antimicrob. Agents Chemother.* **59** (2015).
10. L. Zhou, L. Yang, S. Tilton, J. Wang, Development of a high throughput equilibrium solubility assay using miniaturized shake-flask method in early drug discovery. *J. Pharm. Sci.* **96**, 3052–71 (2007).
11. C. J. Dickson, V. Hornak, D. Bednarczyk, J. S. Duca, Using Membrane Partitioning Simulations To Predict Permeability of Forty-Nine Drug-Like Molecules. *J. Chem. Inf. Model.* **59**, 236–244 (2019).
12. J. D. Irvine, *et al.*, MDCK (Madin-Darby canine kidney) cells: A tool for membrane permeability screening. *J. Pharm. Sci.* **88**, 28–33 (1999).
13. Y. Y. Lau, *et al.*, The use of in vitro metabolic stability for rapid selection of compounds in early discovery based on their expected hepatic extraction ratios. *Pharm. Res.* **19**, 1606–10 (2002).
14. R. S. Obach, Prediction of human clearance of twenty-nine drugs from hepatic microsomal intrinsic clearance data: An examination of in vitro half-life approach and nonspecific binding to microsomes. *Drug Metab. Dispos.* **27**, 1350–9 (1999).
15. L. Di, E. H. Kerns, Y. Hong, H. Chen, Development and application of high throughput plasma stability assay for drug discovery. *Int. J. Pharm.* **297**, 110–9 (2005).
16. T. Wajima, Y. Yano, K. Fukumura, T. Oguma, Prediction of human pharmacokinetic profile in animal scale up based on normalizing time course profiles. *J. Pharm. Sci.* **93**, 1890–900 (2004).
17. I. Mahmood, Prediction of human drug clearance from animal data: application of the rule of exponents and “fu Corrected Intercept Method” (FCIM). *J. Pharm. Sci.* **95**, 1810–21 (2006).
18. I. Mahmood, Interspecies scaling of biliary excreted drugs: a comparison of several methods. *J. Pharm. Sci.* **94**, 883–92 (2005).
